# Targeted resequencing of GWAS loci reveals novel genetic variants for milk production traits

**DOI:** 10.1186/1471-2164-15-1105

**Published:** 2014-12-15

**Authors:** Li Jiang, Xuan Liu, Jie Yang, Haifei Wang, Jicai Jiang, Lili Liu, Sang He, Xiangdong Ding, Jianfeng Liu, Qin Zhang

**Affiliations:** National Engineering Laboratory for Animal Breeding; Key Laboratory of Animal Genetics, Breeding and Reproduction, Ministry of Agriculture of China; College of Animal Science and Technology, China Agricultural University, Beijing, 100193 China

**Keywords:** Genetic variants, Milk production traits, Targeted resequencing

## Abstract

**Background:**

Genome wide association study (GWAS) has been proven to be a powerful tool for detecting genomic variants associated with complex traits. However, the specific genes and causal variants underlying these traits remain unclear.

**Results:**

Here, we used target-enrichment strategy coupled with next generation sequencing technique to study target regions which were found to be associated with milk production traits in dairy cattle in our previous GWAS. Among the large amount of novel variants detected by targeted resequencing, we selected 200 SNPs for further association study in a population consisting of 2634 cows. Sixty six SNPs distributed in 53 genes were identified to be associated significantly with on milk production traits. Of the 53 genes, 26 were consistent with our previous GWAS results. We further chose 20 significant genes to analyze their mRNA expression in different tissues of lactating cows, of which 15 were specificly highly expressed in mammary gland.

**Conclusions:**

Our study illustrates the potential for identifying causal mutations for milk production traits using target-enrichment resequencing and extends the results of GWAS by discovering new and potentially functional mutations.

**Electronic supplementary material:**

The online version of this article (doi:10.1186/1471-2164-15-1105) contains supplementary material, which is available to authorized users.

## Background

Milk production traits are the most economically important traits in dairy cattle. Identification of genetic variants affecting milk production traits is crucial for understanding the genetic mechanisms underlying the phenotypic variation and hence enhancing the breeding efficiency. Although a large number of QTL for milk production traits have been reported [1], merely a few strong candidate genes (including *DGAT1* and *GHR*) [2–6] responsible for the observed effect have been identified.

Genomic selection has been widely implemented in dairy cattle since 2008 and is bringing great changes in dairy cattle breeding system [7–9]. However, gaining better knowledge of the genetic architecture of traits of interest is still important, since this could indeed lead to new insights in the molecular physiology of the interesting phenotypes, and is expected to bring about new opportunities for more effective breeding strategy.

With high throughput SNP genotyping technologies, genome-wide association study (GWAS) has been widely accepted as a primary approach for finding genes relevant to complex traits. Several successful GWAS based on the Illumina BovineSNP50 BeadChip identified a number of important candidate genes associated with milk production traits [10–14]. Although these findings provide new insights into genetic basis of milk production traits, the specific genes and causal variants underlying these traits have not yet been clearly defined because most of the detected SNPs are not the causal variants but markers being potentially in linkage disequilibrium (LD) with the causal variants. Moreover, some of the associated genes or variants do not have known or obvious functions related to milk production traits and some significant SNPs implicate regions with multiple genes or no genes, limiting biological extrapolation.

Recent advances in next generation sequencing (NGS) technologies make it possible to sequence genomic regions exhaustively. Targeted sequencing of specific regions using NGS technologies can efficiently capture all variants in these regions and their potential effects can be assessed by a subsequent association study, which provides an effective approach to find the causal variant affecting the concerned traits [15–19].

In our previous GWAS study in Chinese Holstein cattle, we identified 105 SNPs associated significantly with milk production traits [10]. In this study, we employed the NGS technology to assess the candidate target regions implicated by these SNPs. We then carried out association analysis for the variants revealed by NGS. We further employed the expression analysis for 20 of the significant genes, which could be considered as novel promising candidate functional genes in dairy cattle. Our results provide evidence towards biological function validation of genes for milk production traits in dairy cattle.

## Results

### Discovery of variants using targeted sequencing

The targeted resequencing of DNA of 60 bulls (in 10 pools) yielded large amounts of high-quality sequence data (Additional file 1: Table S1). In total, 112.56 million 100-bp paired-ends reads (22.5 Gb) were obtained from the 10 pools. The sequence data achieved an average coverage of 131.83× per pool, corresponding to an average coverage of 22× per individual. We captured 83.33% of our target regions with > 20 × coverage and 74.15% of target region with >50× coverage, and around 15% of target regions were poorly covered.

These sequences were mapped to the reference Bovine UMD3.1 genome assembly to detect SNPs. A total of 127,218 SNPs (>4x) (Additional file 1: Table S2) were identified, of which 0.53% are novel after comparing with the up to date cattle dbSNP database [20] (Additional file 1: Table S3). The proportion of SNPs which are included in the dbSNP database was consistent across the ten pools, ranging from 99.1% to 99.5%.

To validate the NGS results, Sanger sequencing of PCR amplicons were carried out. We randomly chose three genes for validation. 28 SNPs were discovered by Sanger sequencing, which include all SNPs (18) discovered by NGS in the three genes (Additional file 1: Table S4, Additional file 2: Figure S1). The missing SNPs in NGS were largely due to the fact that the probes designed for NGS failed to cover the entire target regions. Therefore, the NGS results are reliable for further research. These SNPs were categorized by their genic location (exon, UTR, promoter, intron and intergenic regions) and by their predicted effect, including synonymous substitution, non-synonymous substitution and splice site alteration. Notably, among all of the detected SNPs, 735 are located within exonic regions, of which 191 are non-synonymous mutation.

### Association study

From the 127,218 SNPs detected by NGS, we selected 200 SNPs (Additional file 1: Table S5) for association study, including 123 in CDS, 36 in UTR, 33 in promoter regions and 8 in introns. The 200 SNPs were genotyped using Sequenom MassARRAY iPLEX platform in a population of 734 cows, which are daughters of 30 sires. The association analysis showed that a total of 40 SNPs distributed in 33 genes were significantly associated with one or multiple milk production traits (Additional file 1: Table S6). These 40 significant SNPs were located on five chromosomes including BTA1, BTA3, BTA11, BTA14 and BTA20. Among the 33 genes, 17 contain or are close to SNPs with genome-wide significance for milk production traits in our previous GWAS results [10], and five (*DGAT1*, *HEATR7A*, *VPS28*, *CPSF1* and *LOC509113*) have effects on all the five traits.

To enlarge the reference population for association analysis, we imputed the genotypes of the 200 SNPs in another population of 1917 cows, which were half sibs of the 734 cows, based on the Illumina 54 K SNP array genotype data on both populations. Association study was performed again in the combined population consisting of 2634 cows. A total of 66 significant SNPs distributed in 53 genes were identified (Table 1, Additional file 1: Table S7), which include almost all the significant SNPs (38 of 40) from the first association analysis. Of these 53 genes, 26 contain or are close to SNPs with genome-wide significance for milk production traits in our previous GWAS results [10].

**Table 1:** **SNPs significantly associated with milk production traits in the combined population**

SNP-ID	Gene	Amino acid sub	Chr	Position ^a^	Traits ^b^	***P***value ^c^
N7	*PDE9A*		1	144562226	PP	4.68E-05
C8	*DIP2A*	K → Q	1	147894635	PP	1.01E-05
U71	*SLC30A7*		3	42465645	PP	1.13E-04
C30	*SLC30A7*	H → Y	3	42530986	PP	4.88E-06
U36	*STXBP1*		11	98393605	PP	1.43E-05
C204	*EGFL7*	I → M	11	104152101	PP	2.71E-07
U41	*LY6D*		14	1155474	MY,FY,FP,PP	9.62E-05
U42	*LY6H*		14	1449253	MY,PY,FP	5.84E-05
C109	*ZNF34*	H → R	14	1494039	MY,FP,PY,PP	1.74E-04
P18	RPL8*		14	1508300	MY,PY,FP	6.63E-05
S12	*LOC785799*		14	1524572	MY,PY,FP	5.63E-05
P23	GPT*		14	1628421	All	8.08E-08
C110	*PPP1R16A*	A → T	14	1629600	All	2.39E-07
S4	CYHR1*		14	1677522	All	3.14E-08
U46	VPS28*		14	1694862	All	1.99E-09
C111	CPSF1*	T → I	14	1736599	All	1.40E-10
C199	DGAT1*	K → A	14	1802265	All	2.96E-10
U47	HSF1*		14	1806340	FP	2.95E-08
C112	HEATR7A*	Q → R	14	1851040	FP	2.30E-11
C113	HEATR7A*	N → S	14	1878165	All	2.66E-10
U48	LOC509113*		14	1907315	All	2.39E-10
N10	*MAF1*		14	1924112	MY,FP,PP	2.31E-06
P24	*GPAA1*		14	1946673	MY,FP,PP	1.15E-06
P25	*OPLAH*		14	1957462	MY,FP,PP	1.48E-06
C114	*SPATC1*	P → A	14	1977494	MY,FP,PP	1.27E-06
U50	*GRINA*		14	2019072	FP	6.36E-06
C115	*PARP10*	G → D	14	2026781	MY,FP,PP	4.39E-07
C116	*PARP10*	G → S	14	2027812	MY,FP,PP	3.13E-07
C119	LOC786966*	D → A	14	2086893	MY,FP,PP	2.97E-06
C120	LOC786966*	L → G	14	2087763	All	1.66E-07
C125	*EPPK1*	R → Q	14	2138115	PP	9.31E-05
P29	*PUF60*		14	2163044	FP	5.29E-06
P33	LOC506831*		14	2221616	FY,FP,PP	2.29E-04
C127	FAM83H*	V → G	14	2231494	MY,FY,FP,PP	1.08E-04
C128	*MAPK15*	T → M	14	2239085	MY,FY,FP,PP	2.12E-04
C130	*PYCRL*	R → C	14	2308255	PY	2.04E-06
P35	EEF1D*		14	2311270	MY,FP,PP	9.23E-06
P90	EEF1D*		14	2314560	MY,FP,FP	1.21E-04
C132	ZC3H3*	P → L	14	2358243	MY,PY,FP,PP	6.77E-06
C133	ZC3H3*	A → D	14	2358255	MY,PY,FP,PP	8.05E-06
U52	*RHPN1*		14	2462476	MY,FP,FP	1.49E-05
C207	*RHPN1*	T → A	14	2465250	FP,PP	2.29E-04
P89	GPIHBP1*		14	2553652	MY,PY,FP	2.17E-08
C206	*CYP11B1*	A → V	14	2705205	FP	1.57E-10
N8	*CYP11B1*		14	2706012	FY,FP	7.98E-05
C198	*CYP11B1*	E → K	14	2708768	FP	1.87E-05
C136	*GML*	Q → P	14	2715807	FP	1.57E-09
C137	*LYNX1*	L → F	14	2816429	MY,FP,PP	7.22E-05
S8	*GPR20*		14	3640627	MY,FP,PP	8.90E-05
C138	PTK2*	I → M	14	4061098	MY,FY,FP	3.16E-05
P48	EIF2C2*		14	4129075	MY,FY,FP	1.62E-04
C139	TRAPPC9*	C → G	14	4352117	MY,PY,FP	3.45E-07
C140	TRAPPC9*	G → R	14	4472220	MY,FY,FP	1.29E-04
U70	*KCNK9*		14	4743187	MY,FP	6.83E-05
C147	*FAM135B*	M → V	14	5603441	MY,FY,PP	1.24E-04
C194	GHR*	F → Y	20	31909476	MY,FY,PP	6.44E-09
P63	*PLCXD3*		20	33027949	FP,PP	1.96E-04
U60	*PLCXD3*		20	33229971	PP	7.47E-07
C163	*C6*	P → L	20	33376024	MY,FP,PP	3.34E-05
C168	*C7*	T → I	20	33578727	MY,FP,PP	8.33E-05
C169	*C7*	T → M	20	33582457	PP	2.81E-09
C171	*DAB2*	A → V	20	35073744	FP,PP	1.88E-05
C175	*OSMR*	R → M	20	35544340	PP	1.56E-05
C176	*OSMR*	M → L	20	35561705	PP	5.21E-05
P72	*GDNF*		20	36634182	FP,PP	4.81E-07
C184	NIPBL*	I → V	20	37238542	FP,PP	1.97E-06

a: based on the UMD_3.1 genome assembly.

b: MY = milk yield, FY = fat yield, PY = protein yield, FP = fat percentage, PP = protein percentage

c: These values were from the combined population. For SNPs whichwere significant for more than one trait, the maximum *P* values are presented.

*: These genes were selected for mRNA expression analysis.

### Expression analysis of the candidate genes

**Figure 1:**
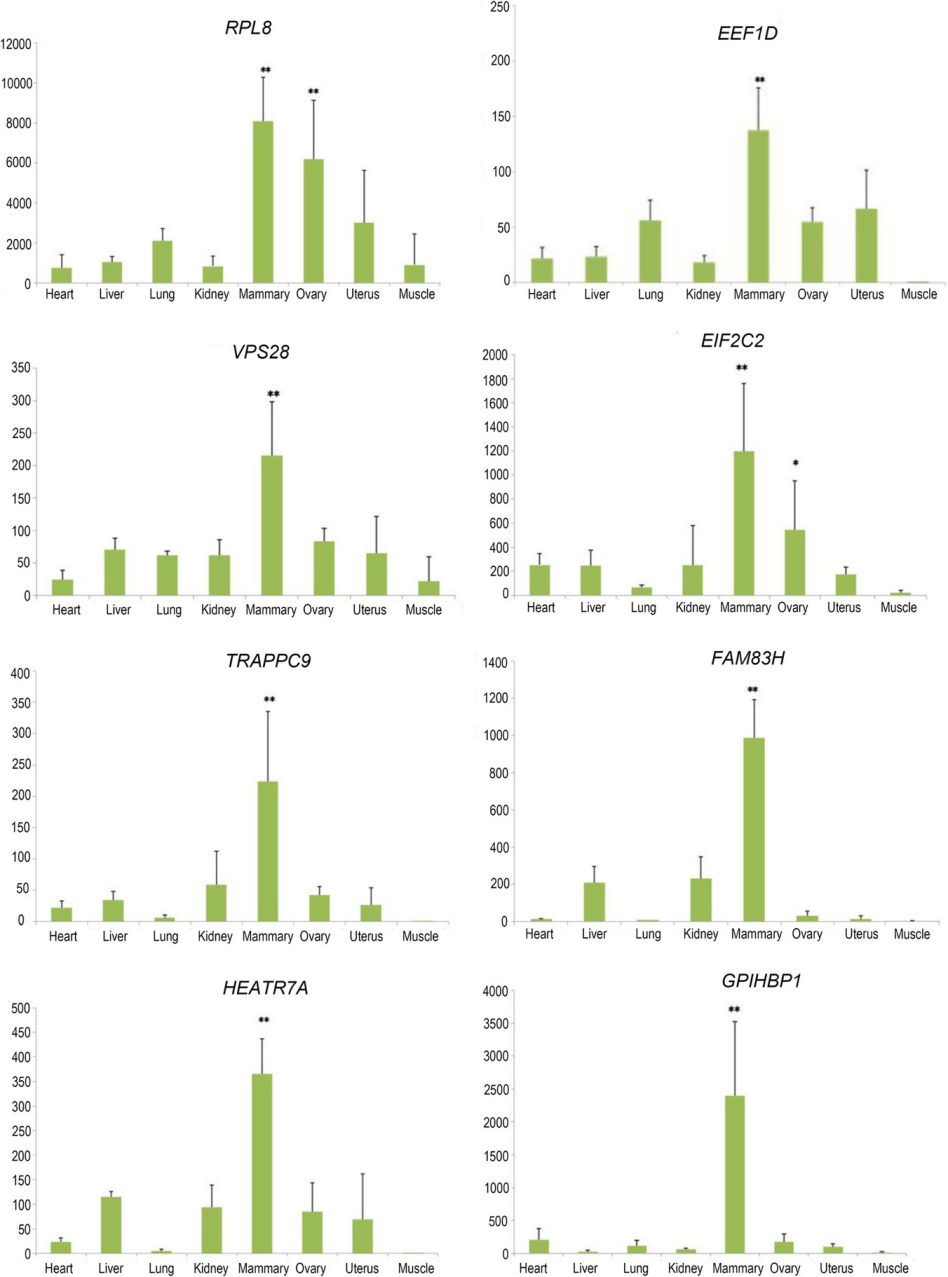
**Relative mRNA expression of eight genes in eight tissues of four lactating cows.** Three replicates were performed for each reaction.

We chose 20 out of the 53 significant genes to analyze their mRNA expression in eight different tissues of lactating cows. Fifteen of them showed higher mRNA expression level in mammary gland than in the other seven tissues, especially *RPL8*, *EEF1D*, *VPS28*, *EIF2C2*, *TRAPPC9*, *FAM83H*, *HEATR7A* and *GPIHBP1* (Figure 1, Additional file 2: Figure S2), and all of them had the lowest expression level in muscle (Additional file 2: Figure S2). Notably, the two genes *DGAT1* and *GHR*, which have been functionally confirmed to have large effects on milk production traits from previous studies [2–4], had higher mRNA expression in liver besides in mammary gland (Figure 1). Furthermore, *EEF1D* and *RPL8* showed the highest mRNA expression levels in mammary gland among all the 20 genes (Additional file 1: Table S8) and *GPIHBP1* showed the largest difference in mRNA expression between in mammary gland and in the other seven tissues.

## Discussion

Recent advances in next-generation sequencing (NGS) technology provide a cost-effective approach for large-scale resequencing of target genomic regions to identify causal variants. We describe here a pooled NGS study for resequencing of target regions containing 167 genes which were found to be potentially associated with milk production traits in our previous study [10]. We then carried out association analysis for 200 novel SNPs revealed by NGS in these regions. A total of 66 SNPs located on five chromosomes (BTA1, BTA3, BTA11, BTA14 and BTA20) and distributed in 53 genes were found to be significantly associated with one or multiple milk production traits.

In this study, estimated breeding values (EBVs) of the studied traits were used as trait scores for the association analysis. Some recent study [21] showed that EBVs estimated using familial data should not be used as trait score for association analysis because this may lead to high false-positive rate. The authors recommended that when each genotyped individual has its own associated trait score(s), the “measured genotype” approach, in which the phenotypic value is used as a trait score and the additive polygenic effects are included in the model to account for the familial relatedness of individuals in the pedigree using the additive genetic relationships among individuals. However, for milk production traits in dairy cattle, it is hard to use the phenotypic value as a trait score because there are repeated measurements on each individual. Another problem of using phenotypic value is that the systematic environmental effects on measured phenotypes may not be efficiently corrected because the sample size for association study is usually relative small in comparison with that for EBV prediction. These may be the reasons why in all GWAS studies in dairy cattle either EBVs or de-regressed EBVs were used as trait score. In our previous GWAS in Chinese Holstein [10], we also compared using EBVs and de-regressed EBVs as phenotypes for our GWAS and it turned out that the findings of them were basically overlap. Therefore, in this study we chose to use EBVs as trait scores for association analysis, and we included the residual polygenic effects in the model to account for the familial relatedness of individuals.

The majority of the significant SNPs (49 out of 66) are located on BTA14. These SNPs are distributed in 40 genes, of which 32 were related to more than one trait, including 9 (*DGAT1, LOC786966, PPP1R16A, CPSF1, HEATR7A, LOC509113, CYHR1, VPS28* and *GPT*) related to all the five traits. BTA14 has been reported repeatedly to harbor a large number of genetic variants associated with milk production traits in dairy cattle [14, 22–24]. In addition to numerous QTL [1], a few candidate genes were also reported. In addition to the *DGAT1* gene, which was confirmed in almost all association studies to have strong association with multiple milk production traits [3, 4], a number of other genes were also reported, including *MAPK15* [25] *, CYP11B1* [25], *VPS28* [13], *GPIHBP1* [13] *, KCNK9* [13, 25], *TRAPPC9* [13] and *CYHR1* [13]. All these genes were confirmed in the present study. Since such a large number of significant SNPs are located on BAT14, it is very likely that some of the effects are due to linkage disequilibrium (LD) with the real causative variants. We analyzed the LD levels between all the significant SNPs. The results show that some of them are in LD with *r*^2^ greater than 0.2 (Additional file 2: Figure S3). In particular, several of them are in strong LD with the SNP within *DGAT1* (SNP C199), which is the most significant SNP, and their significance are strongly correlated their LD levels with C199 (Additional file 1: Table S12). We then conducted a further association analysis for the SNPs on BTA14 with C199 fixed in the model. It turned out that most of the SNPs became non-significant (in particular the SNPs with strong LD with C199) or less significant (Additional file 1: Table S12, taking the trait fat percentage as an example). These results indicate that the effects of these SNPs revealed by the original association analysis (without C199 in the model) are indeed fully or partly due to their LD with C199.

Eleven out of the 66 significant SNPs are located on BTA20 in 8 genes. All of them were associated with protein percentage and seven were also related to fat percentage, of which three were also related to milk yield. Many researches indicated the importance of BTA20 with respect to milk production traits in dairy cattle [13, 14, 26, 27]. In particular, the *GHR* gene on BTA20 was proved to be an important candidate gene for milk production traits by several studies [2, 14, 28]. Furthermore, for protein percentage, many QTL were identified on BTA20 [1], and enrichment of significant SNPs on BTA20 was also reported [29]. Our results were consistent with these findings.

On BTA1, BTA3 and BTA11, we identified two SNPs associated with milk protein percentage. The two SNPs on BTA1 are within *PDE9A* and *DIP2A*, respectively. Both SNPs on BTA3 are within *SLC30A7*. The two SNPs on BTA11 are within *EGFL7* and *STXBP1*, respectively. None of these genes have been reported to be associated with protein percentage before, although some QTL [1] as well as significant SNPs [13, 14, 23] were reported on these chromosomes.

To further explore the potential functions of the genes revealed in this study, we analyzed the mRNA expression of 20 significant genes in eight tissues of lactating dairy cows. It turned out that 15 genes had the highest mRNA expression level in mammary gland compared to other tissues, indicating that these genes might play important roles during lactation period in dairy cattle. In particular, the expression levels of *RPL8* and *EEF1D* in mammary gland were the highest among the 20 genes (Additional file 1: Table S8), both of which were very strongly associated with fat percentage (*P* = 2.26 × 10^-15^ and 2.07 × 10^-15^, respectively) in the association analysis. Maningat et al. [30] investigated gene expression in human mammary epithelium during lactation and found that many ribosomal protein family members, including *RPL8*, showed the highest expression level in milk fat globule. Pisanu et al. [31] found that *EEF1D* was specifically expressed in milk fat globule in sheep. These findings further support the association of these two genes with fat percentage. Furthermore, the *GPIHBP1* gene, which was also very strongly associated with fat percentage (*P* = 5.00 × 10^-18^), showed the largest difference in mRNA expression level between mammary gland and the other seven tissues. The GPIHBP1 protein is the glycosylphosphatidylinositol (GPI)-anchored protein of the lymphocyte antigen 6 (Ly6) family and is essential in the lipolytic processing of triglycerides within chylomicrons [32–34]. This suggests that *GPIHBP1* may be involved in the process of milk fat production.

## Conclusions

In summary, we detected a number of novel variants from significant regions associated with milk production traits in our previous GWAS by NGS technology. The association analysis of 200 important variants revealed 66 significant SNPs distributed in 53 genes associated with milk production traits. The expression analysis for 20 of the 53 genes identified 15 genes that were specifically highly in mammary gland and may contribute to milk production traits. Further study and integration of these findings will surely promote a better understanding of the global genetic architecture of milk production traits in dairy cattle.

## Methods

### Ethics statements

The whole procedure for collection of the tissue samples of all animals was carried out in strict accordance with the protocol approved by the Animal Welfare Committee of China Agricultural University (Permit number: DK996).

### DNA preparation and pooling

DNA was extracted from semen samples of 60 bulls. The semen samples were digested using proteinase K for 4 to 6 hours, and genomic DNA was extracted by using the standard phenol/chloroform method. The extracted DNA was assessed on an agarose gel and spectrophotometer for quality testing and then quantified using the Illumina Eco Real-Time PCR System. Ten pools were constructed with each pool containing normalized DNA of 6 bulls of equimolar amounts.

### Capture of target regions and next generation DNA sequencing

Candidate genes were selected which harbor or are closest to at least one SNP which was shown to be significantly associated with milk production traits with *P* values less than 10^-5^ in our previous GWAS [10]. For each of these genes, a target region was defined such that it comprised the entire gene and its promoter region within 3 kb upstream. Some regions may harbor more than one gene if the genes are close to each other. The sizes of the target regions ranged from 4 kb to 937 kb with an average of 72.5 kb. A total of 91 regions were captured and they were distributed on chromosomes 1, 3, 5, 6, 8, 9, 10, 11, 14, 18, 20, 26, and X. The cRNA oligonulceotide baits for these targets were designed using Agilent’s web-based bait design tool (https://earray.chem.agilent.com/earray/) based on the Btau4.0 bovine genome assembly. This custom capture platform includes 6.6 Mb targeted features (SureSelect Target Enrichment Kit). Genomic DNA was captured by hybridization in solution to the designed baits [35] following the manufacture’s protocols (Agilent Technologies). Library construction and sequencing were performed according to manufacturer’s protocols. Sequencing was carried out on an Illumina HiSeq 2000.

### Sequence data analysis

Sequence data were processed through Illumina pipeline v1.6 using default parameters. Reads of 100 bp were aligned to the bovine reference genome sequence (UMD3.1 bovine genome assembly) using the BWA algorithms [36] and further processed using the SAMtools software [37]. Sequencing depth of coverage was defined as the number of sequencing reads, which had been filtered and mapped. For each pool, the percentage of target regions covered by more than 50× reads ranged from 70.27% to 77.57% with an average of 74.15%. High-confidence single-base pair variants were detected in each pool using BWA with a minimum of 4 high-quality aligned reads (base quality ≥ 20). Indels were identified from within unaligned reads, which were also supported by >4 unaligned reads that contained an insertion/deletion.

To prioritize a variant, variants were annotated according to their location within the target regions based on the genome annotation downloaded from NCBI (UMD3.1 bovine genome assembly), including (i) present in coding regions (missense variant at an amino acid); (ii) present in 5’UTR, 3’UTR or promoter region; (iii) present at a splice site (two bases upstream or downstream the intron-exon boundary); (iv) coding idel; and (v) nonsense variant. We also assessed our results by comparing with cattle SNPs in the dbSNP database of NCBI based on the UMD3.1 genome builds.

### Validation of SNPs by Sanger sequencing

A total of 64 primer pairs (Additional file 1: Table S9) were designed to validate SNPs of three genes including *GHR*, *PDE9A* and *NOTCH1*. These primers covered all coding regions and their flanking intron sequences. A DNA pool was constructed from ten randomly selected bulls (50 ng/μL per sample). SNPs were validated by Sanger sequencing using ABI 3730XL.

### Genotyping

We chose 200 SNPs to genotype for association analysis according to the following procedure. First, we selected missense mutations within the target genes. Second, if there is no missense mutation, we selected SNPs in 5’UTR or 3’UTR. Third, if there is no polymorphism in 5’UTR or 3’UTR, SNPs at splice sites or in promoter region were selected. Finally, we chose SNPs in intron otherwise. These SNPs were assayed in whole-genome-amplified DNA of 734 cows using the Sequenom MassARRAY iPLEX genotyping technology [38]. These cows were distributed in 30 sire families (Additional file 1: Table S10) and were from 30 Holstein cattle farms in Beijing and Shanghai in China, where regular and standard performance testing (dairy herd improvement, DHI) has been conducted since 1999. All SNPs were amplified in multiplexed pools of 25–28 assays, using 10 ng of template DNA from each sample. All the primers were designed by AssayDesigner v.3.1 software. SpectroCHIPs with 384-wells were analyzed by a MassArray MALDI-TOF Compact system with a solid phase laser mass spectrometer. The resulting spectra were called and analyzed by the SpectroTyper v.4.0 software. We obtained high quality data (Call rate >90%, MAF >1%) in all samples.

### Genotype imputation

To enlarge the reference population for association analysis, we imputed the genotypes of the 200 SNPs in another 1917 cows which are half sibs of the 717 cows genotyped using Sequenom MassARRAY iPLEX as mentioned above. Both samples of cows had been genotyped with the Illumina 54 K bovine SNP arrays. The imputation was carried out using the BEAGLE software [39]. The combined reference population after imputation had 2634 individuals for association analysis of the 200 SNPs.

### Association analysis

We performed association analysis between the 200 SNPs and five milk production traits (milk yield, fat yield, protein yield, fat percentage, and protein percentage). For each SNP and each trait the analysis was carried out based on the following model:


Where **y** is the vector of estimated breeding values (EBVs) of the trait of all individuals, *μ* is the overall mean, **x** is the vector of the SNP genotype indicators with values 0, 1 or 2 corresponding to genotypes 11, 12 and 22 (assuming 2 is the allele with a minor frequency), respectively, *b* is the regression coefficient, **a** is the vector of the residual polygenetic effects with  (where **A** is the additive genetic relationship matrix, which was calculated based on the full pedigree containing a total of 8344 individuals, and  is the additive genetic variance), **Z** is the design matrix of **a**, and **e** is the vector of residual errors with . The estimate of *b* and its corresponding sampling variance *Var*(*b*) were obtained via the mixed model equations (MME) corresponding to the model, and a Wald Chi-squared statistic  with *df* = 1 was constructed to examine whether the SNP is significantly associated with the trait.

The Bonferroni method was adopted to adjust for multiple testing from the number of SNPs tested, and the Bonferroni corrected *P* value to declare significance was *P* value <0.05/*N*, where *N* is the number of SNPs tested.

### Expression analysis of candidate genes

Four Chinese Holstein cows which were in the same period of lactation (around 350 days in milk) were selected. Eight tissues samples (heart, liver, lung, kidney, mammary gland, ovary, uterus and muscle) from each cow were collected within 30 min after slaughter and stored at liquid nitrogen. Expression levels of selected candidate genes in the eight tissues were performed using real time quantitative PCR. The total RNA was extracted from each sample and was reversely transcribed to cDNA in a 40 μL reaction using the PrimeScript RT reagent Kit (Takara Biotechnology, Tokyo, Japan). We designed the qPCR primers based on the reference sequence in NCBI (Additional file 1: Table S11) using the Primer 3 web-tool (http://frodo.wi.mit.edu/primer3/) and the Oligo 6.0 software (Molecular Biology Insights Inc., Cascade, CO). Amplification efficiencies of all primers were calculated based on the standard curves. PCR amplifications were performed in a final volume of 20 μL which consisted of 1 μL cDNA, 1 μL (10pM/μL) of both forward primer and reverse primer, 10 μL of Master Mix (2×) and water (Roche Applied Science). All RT-PCR reactions of each sample were run in triplicate and the mRNA expression of each gene in each tissue was measured relative to the housekeeping gene glyceraldehyde phosphate dehydrogenase (*GAPDH*) in the sample.

### Availability of supporting data

The whole SNP data revealed by the target sequencing are available in the dbSNP database [http://www.ncbi.nlm.nih.gov/projects/SNP/snp_viewTable.cgi?handle=CAU_QZHANG]. The other data sets supporting the results of this article are included within the article and its additional files.

## Electronic supplementary material

Additional file 1: Table S1: Yield of the next generation sequencing in 10 libraries. **Table S2.** Number of high-quality SNPs in 10 libraries. **Table S3.** Novel and known SNPs in the 10 libraries. **Table S4.** SNPs detected in *NOTCH1*, *PDE9A* and *GHR* by Sanger sequencing. **Table S5.** List of 200 SNPs selected for association analysis. **Table S6.** SNPs significantly associated with milk production traits in the Sequenom genotyped population. **Table S7.** SNPs significantly associated with milk production traits in the combined population (Sequenom genotyped + imputed population). **Table S8.** Relative expression of the 20 significant genes in mammary gland. **Table S9.** Primers designed for Sanger sequencing of *NOTCH1*, *PDE9A* and *GHR.* **Table S10.** Distribution of daughters in 30 sire families for association analysis. **Table S11.** Primers for real time RT-PCR of 20 significant genes. **Table S12.** LD levels (*r* ^2^) between SNPs on BTA14 and the *DGAT1* SNP (C199) and *P* values of association analysis for fat percentage using models without and with C199 fixed. (DOC 714 KB)

Additional file 2: Figure S1: DNA sequencing chromatogram of SNPs detected in *NOTCH1*, *PDE9A* and *GHR* for validation of NGS results. **Figure S2.** Relative mRNA Expression of 20 significant genes in eight tissues of four cows by real time RT-PCR. **Figure S3.** Linkage disequilibrium levels (*r* ^2^) between the significant SNPs on BTA14 obtained by using Haploview. (DOC 10 MB)
