# Supplementary material for: Targeted resequencing of GWAS loci reveals novel genetic variants for milk production traits
Source: BMC Genomics. 2014 Dec 15;15(1):1105. doi: 10.1186/1471-2164-15-1105 (PMC4377845; doi:10.1186/1471-2164-15-1105)
Supplement: Supplementary file 1 — Additional file 1: Table S1: Yield of the next generation sequencing in 10 libraries. Table S2. Number of high-quality SNPs in 10 libraries. Table S3. Novel and known SNPs in the 10 libraries. Table S4. SNPs detected in NOTCH1, PDE9A and GHR by Sanger sequencing. Table S5. List of 200 SNPs selected for association analysis. Table S6. SNPs significantly associated with milk production traits in the Sequenom genotyped population. Table S7. SNPs significantly associated with milk production traits in the combined population (Sequenom genotyped + imputed population). Table S8. Relative expression of the 20 significant genes in mammary gland. Table S9. Primers designed for Sanger sequencing of NOTCH1, PDE9A and GHR. Table S10. Distribution of daughters in 30 sire families for association analysis. Table S11. Primers for real time RT-PCR of 20 significant genes. Table S12. LD levels (r 2) between SNPs on BTA14 and the DGAT1 SNP (C199) and P values of association analysis for fat percentage using models without and with C199 fixed. (DOC 714 KB) [file 12864_2014_6834_MOESM1_ESM.doc]

Additional file 1: Table S1 Yield of the next generation sequencing in 10 libraries

| Sample Info | Total Rds | Rds Len | Insert Size | Yield(bps) |
| --- | --- | --- | --- | --- |
| M1 | 7,850,315 | 100 | 180 | 1,570,063,000 |
| M2 | 10,580,631 | 100 | 180 | 2,116,126,200 |
| M3 | 9,409,162 | 100 | 180 | 1,881,832,400 |
| M4 | 12,877,070 | 100 | 180 | 2,575,414,000 |
| M5 | 13,045,405 | 100 | 180 | 2,609,081,000 |
| M6 | 9,781,129 | 100 | 180 | 1,956,225,800 |
| M7 | 12,206,342 | 100 | 180 | 2,441,268,400 |
| M8 | 12,869,749 | 100 | 180 | 2,573,949,800 |
| M9 | 14,160,205 | 100 | 180 | 2,832,041,000 |
| M10 | 9,782,521 | 100 | 180 | 1,956,504,200 |
| Total | 112,562,529 |  |  | 22,512,505,800 |

Additional file 1: Table S2 Number of high-quality SNPs in 10 libraries

| Libraries | >4x | >20x | >30x |
| --- | --- | --- | --- |
| M1 | 47261 | 17214 | 10399 |
| M2 | 55749 | 32030 | 24800 |
| M3 | 54751 | 29463 | 22275 |
| M4 | 66030 | 39744 | 30873 |
| M5 | 54579 | 32031 | 25017 |
| M6 | 58590 | 32868 | 24937 |
| M7 | 61321 | 36157 | 27972 |
| M8 | 62742 | 37349 | 29069 |
| M9 | 63911 | 39363 | 30931 |
| M10 | 54433 | 30184 | 23349 |

Additional file 1: Table S3 Novel and known SNPs in the 10 libraries

| Libraries | >4x | novel | known |
| --- | --- | --- | --- |
| M1 | 47261 | 399 | 46862 |
| M2 | 55749 | 263 | 55486 |
| M3 | 54751 | 257 | 54494 |
| M4 | 66030 | 329 | 65701 |
| M5 | 54579 | 296 | 54283 |
| M6 | 58590 | 284 | 58306 |
| M7 | 61321 | 337 | 60984 |
| M8 | 62742 | 290 | 62452 |
| M9 | 63911 | 312 | 63599 |
| M10 | 54433 | 268 | 54165 |

Additional file 1: Table S4 SNPs detected in *NOTCH1*, *PDE9A* and *GHR* by Sanger sequencing

| Gene | SNP-ID | Position | Alleles | Region | Detected in NGS |
| --- | --- | --- | --- | --- | --- |
| NOTCH1 | NOT1 | chr11 104028662 | C/A | intron | No |
| NOTCH1 | NOT2 | chr11 104011816 | A/G | CDS | No |
| NOTCH1 | NOT3 | chr11 104011762 | T/C | CDS | No |
| NOTCH1 | NOT4 | chr11 104003041 | A/G | CDS | Yes |
| NOTCH1 | NOT5 | chr11 104002890 | A/G | Intron | Yes |
| NOTCH1 | NOT6 | chr11 104002731 | A/G | CDS | Yes |
| NOTCH1 | NOT7 | chr11 103999363 | T/C | CDS | Yes |
| NOTCH1 | NOT8 | chr11 103999345 | T/C | CDS | Yes |
| NOTCH1 | NOT9 | chr11 103999270 | C/T | CDS | Yes |
| NOTCH1 | NOT10 | chr11 103996622 | A/G | intron | Yes |
| PDE9A | PDE1 | chr1 144525732 | C/A | intron | No |
| PDE9A | PDE2 | chr1 144562226 | G/A | intron | Yes |
| PDE9A | PDE3 | chr1 144572284 | G/A | intron | No |
| PDE9A | PDE4 | chr1 144587013 | G/A | intron | Yes |
| PDE9A | PDE5 | chr1 144591176 | G/A | intron | No |
| PDE9A | PDE6 | chr1 144592409 | C/T | intron | Yes |
| PDE9A | PDE7 | chr1 144592421 | G/A | intron | No |
| PDE9A | PDE8 | chr1 144597205 | C/T | intron | No |
| PDE9A | PDE9 | chr1 144597336 | A/G | CDS | Yes |
| PDE9A | PDE10 | chr1 144597491 | G/A | intron | Yes |
| PDE9A | PDE11 | chr1 144599244 | G/T | CDS | Yes |
| PDE9A | PDE12 | chr1 144601832 | G/A | CDS | No |
| GHR | GHR1 | chr20 31911609 | A/G | intron | Yes |
| GHR | GHR2 | chr20 31909476 | T/A | CDS | No |
| GHR | GHR3 | chr20 31902571 | G/T | intron | Yes |
| GHR | GHR4 | chr20 31902448 | G/A | intron | Yes |
| GHR | GHR5 | chr20 31891788 | A/G | intron | Yes |
| GHR | GHR6 | chr20 31891025 | C/T | CDS | Yes |

Additional file 1: Table S5 List of 200 SNPs selected for association analysis

| SNP-ID | Chr | position | alleles | gene | dbSNP-ID | region/type | aasub |
| --- | --- | --- | --- | --- | --- | --- | --- |
| N2 | 9 | 6678207 | CA | *LOC100300749* |  | intron |  |
| N3 | 9 | 8862464 | CG | *LMBRD1* |  | intron |  |
| N5 | 9 | 20300187 | CT | *BCKDHB* |  | intron |  |
| N7 | 1 | 144562226 | GA | *PDE9A* |  | intron |  |
| N8 | 14 | 2706012 | GC | *CYP11B1* |  | intron |  |
| N10 | 14 | 1924112 | CG | *MAF1* |  | intron |  |
| N11 | 1 | 144598855 | AG | *PDE9A* |  | intron |  |
| N12 | 1 | 144597491 | GA | *PDE9A* |  | intron |  |
| C3 | 1 | 58757076 | AG | *SIDT1* |  | ns | M→V |
| C4 | 1 | 58768744 | GA | *SIDT1* |  | ns | R→Q |
| C5 | 1 | 140924060 | GA | *SETMAR* | rs135375820 | ns | R→H |
| C7 | 1 | 147891118 | GA | *DIP2A* | rs43251570 | ns | R→H |
| C8 | 1 | 147894635 | AC | *DIP2A* | rs43251562 | ns | K→Q |
| C11 | 1 | 150250457 | TC | *DOPEY2* | rs134489759 | ns | S→P |
| C12 | 1 | 150250784 | AG | *DOPEY2* | rs110665253 | ns | T→A |
| C17 | 1 | 152885633 | CA | *ETS2* | rs108955011 | ns | L→I |
| C18 | 1 | 153301784 | TC | *PIK3R4* | rs109812773 | ns | C→R |
| C20 | 2 | 10762465 | CT | *FSIP2* | rs137768151 | ns | R→G |
| C22 | 3 | 26438257 | CG | *PTGFRN* | rs109403878 | ns | R→P |
| C23 | 3 | 26438323 | TC | *PTGFRN* | rs109625522 | ns | K→R |
| C25 | 3 | 26593559 | GA | *CD2* | rs136618819 | ns | T→M |
| C26 | 3 | 26593575 | GC | *CD2* | rs133131840 | ns | Q→E |
| C27 | 3 | 26765231 | GA | *IGSF3* | rs137143925 | ns | V→I |
| C28 | 3 | 26769804 | GA | *IGSF3* |  | ns | V→I |
| C29 | 3 | 27014362 | GA | *ATP1A1* |  | ns | R→W |
| C30 | 3 | 42530986 | GA | *SLC30A7* | rs110456593 | ns | H→Y |
| C31 | 3 | 42935165 | CT | *CDC14A* |  | ns | A→T |
| C32 | 3 | 48605960 | CG | *ALG14* | rs135127345 | ns | I→M |
| C33 | 3 | 57421819 | CT | *SH3GLB1* |  | ns | A→T |
| C35 | 3 | 60357800 | AG | *TTLL7* | rs42447625 | ns | I→V |
| C36 | 3 | 60411415 | GA | *TTLL7* | rs109065165 | ns | V→I |
| C39 | 3 | 80097504 | CG | *LEPR* |  | ns | R→S |
| C40 | 3 | 80104102 | TC | *LEPR* |  | ns | S→G |
| C43 | 3 | 108623744 | GC | *INPP5B* | rs110236970 | ns | L→F |
| C47 | 5 | 25023942 | GC | *FGD6* | rs110002713 | ns | F→L |
| C48 | 5 | 25024049 | TC | *FGD6* | rs110802737 | ns | M→V |
| C51 | 5 | 45133605 | AG | *CPM* | rs109475249 | ns | T→A |
| C52 | 5 | 51254695 | CG | *MON2* |  | ns | Q→H |
| C54 | 5 | 75659419 | GC | *NCF4* |  | ns | E→Q |
| C55 | 5 | 75659489 | AG | *NCF4* |  | ns | K→R |
| C58 | 5 | 75737606 | GA | *CSF2RB* |  | ns | D→N |
| C59 | 5 | 75744795 | CT | *CSF2RB* |  | ns | P→L |
| C60 | 5 | 83597494 | TC | *ITPR2* |  | ns | Y→H |
| C61 | 5 | 83895731 | GA | *ITPR2* |  | ns | A→T |
| C65 | 5 | 114100015 | AG | *LOC786131* | rs137407445 | ns | I→M |
| C68 | 6 | 23453676 | CT | *MANBA* |  | ns | R→W |
| C69 | 6 | 23535986 | GC | *MANBA* | rs109912202 | ns | E→Q |
| C70 | 6 | 38007713 | GA | *ABCG2* | rs109912292 | ns | R→H |
| C71 | 6 | 38009089 | AG | *ABCG2* | rs111021087 | ns | Y→C |
| C73 | 6 | 94935113 | CG | *FRAS1* | rs110224127 | ns | L→V |
| C74 | 8 | 36600447 | CT | *PTPRD* |  | ns | T→M |
| C75 | 9 | 75191551 | AG | *PDE7B* |  | ns | I→V |
| C76 | 9 | 75560882 | CT | *MAP3K5* | rs109478031 | ns | D→N |
| C78 | 11 | 7324222 | TG | *SLC9A2* | rs135352549 | ns | S→A |
| C79 | 11 | 7414498 | GA | *SLC9A2* | rs133616587 | ns | G→S |
| C81 | 11 | 92297814 | CA | *GGTA1* |  | ns | A→S |
| C82 | 11 | 92302776 | CT | *GGTA1* |  | ns | V→I |
| C84 | 11 | 98406276 | GA | *C11H9orf117* |  | ns | R→Q |
| C87 | 11 | 101280548 | AG | *LAMC3* | rs132760961 | ns | Q→R |
| C88 | 11 | 101284517 | AG | *LAMC3* | rs135170603 | ns | Q→R |
| C98 | 11 | 101366845 | GT | *NUP214* |  | ns | G→V |
| C99 | 11 | 103047377 | TC | *GFI1B* | rs17871660 | ns | L→P |
| C100 | 11 | 103303475 | GA | *LGB* | rs110066229 | ns | G→D |
| C101 | 11 | 103304757 | TC | *LGB* | rs109625649 | ns | V→A |
| C104 | 11 | 103329481 | CT | *LCN9* | rs109318024 | ns | R→C |
| C105 | 11 | 103422400 | GA | *KCNT1* |  | ns | A→T |
| C107 | 11 | 104003041 | TC | *NOTCH1* | rs109811642 | ns | I→V |
| C108 | 11 | 104015686 | CT | *NOTCH1* |  | ns | V→M |
| C109 | 14 | 1494039 | TC | *ZNF34* | rs110226383 | ns | H→R |
| C110 | 14 | 1629600 | CT | *PPP1R16A* |  | ns | A→T |
| C111 | 14 | 1736599 | CT | *CPSF1* | rs134432442 | ns | T→I |
| C112 | 14 | 1851040 | TC | *HEATR7A* |  | ns | Q→R |
| C113 | 14 | 1878165 | TC | *HEATR7A* |  | ns | N→S |
| C114 | 14 | 1977494 | GC | *SPATC1* | rs133219746 | ns | P→A |
| C115 | 14 | 2026781 | GA | *PARP10* | rs136591563 | ns | G→D |
| C116 | 14 | 2027812 | GA | *PARP10* | rs110228339 | ns | G→S |
| C119 | 14 | 2086893 | CT | *LOC786966* |  | ns | D→A |
| C120 | 14 | 2087763 | GA | *LOC786966* |  | ns | L→G |
| C122 | 14 | 2133441 | TC | *EPPK1* |  | ns | F→S |
| C125 | 14 | 2138115 | GA | *EPPK1* |  | ns | R→Q |
| C127 | 14 | 2231494 | TG | *FAM83H* |  | ns | V→G |
| C128 | 14 | 2239085 | GA | *MAPK15* | rs110323635 | ns | T→M |
| C129 | 14 | 2267378 | AC | *ZNF623* | rs109209237 | ns | V→G |
| C130 | 14 | 2308255 | CT | *PYCRL* |  | ns | R→C |
| C131 | 14 | 2308708 | CT | *PYCRL* |  | ns | R→C |
| C132 | 14 | 2358243 | CT | *ZC3H3* | rs111025419 | ns | P→L |
| C133 | 14 | 2358255 | CA | *ZC3H3* | rs110446729 | ns | A→D |
| C134 | 14 | 2512322 | CA | *ZNF696* | rs110588121 | ns | G→T |
| C135 | 14 | 2512650 | GA | *ZNF696* |  | ns | T→M |
| C136 | 14 | 2715807 | TG | *GML* | rs41714067 | ns | Q→P |
| C137 | 14 | 2816429 | CT | *LYNX1* | rs108952604 | ns | L→F |
| C138 | 14 | 4061098 | TG | *PTK2* |  | ns | I→M |
| C139 | 14 | 4352117 | TG | *TRAPPC9* | rs132688804 | ns | C→G |
| C140 | 14 | 4472220 | GC | *TRAPPC9* | rs109850318 | ns | G→R |
| C144 | 14 | 5268458 | GT | *COL22A1* | rs110217491 | ns | A→S |
| C147 | 14 | 5603441 | GA | *FAM135B* | rs109575847 | ns | M→V |
| C148 | 14 | 7441087 | CA | *KHDRBS3* |  | ns | A→S |
| C153 | 18 | 60360276 | TG | *MGC137725* | rs42558829 | ns | T→P |
| C154 | 18 | 64014683 | CT | *ZNF71* | rs110986280 | ns | A→L |
| C155 | 18 | 64619056 | CA | *ZNF805* |  | ns | T→A |
| C156 | 18 | 64670471 | GA | *ZNF264* | rs110636652 | ns | A→H |
| C157 | 20 | 28364930 | TC | *PARP8* | rs133600728 | ns | T→A |
| C158 | 20 | 28395737 | AG | *PARP8* |  | ns | L→S |
| C159 | 20 | 31891050 | TC | *GHR* | rs109300983 | ns | S→G |
| C160 | 20 | 31909478 | AT | *GHR* |  | ns | F→Y |
| C163 | 20 | 33376024 | CT | *C6* |  | ns | P→L |
| C168 | 20 | 33578727 | GA | *C7* | rs135330647 | ns | T→I |
| C169 | 20 | 33582457 | GA | *C7* |  | ns | T→M |
| C171 | 20 | 35073744 | CT | *DAB2* | rs134053035 | ns | A→V |
| C172 | 20 | 35249040 | CT | *FYB* | rs109262355 | ns | A→V |
| C173 | 20 | 35249313 | CT | *FYB* |  | ns | P→L |
| C175 | 20 | 35544340 | CA | *OSMR* | rs41580312 | ns | R→M |
| C176 | 20 | 35561705 | TA | *OSMR* | rs41947101 | ns | M→L |
| C177 | 20 | 35586360 | AG | *LOC100138964* | rs110115552 | ns | H→R |
| C180 | 20 | 36020484 | GA | *EGFLAM* | rs41941623 | ns | R→C |
| C181 | 20 | 36024415 | TC | *EGFLAM* | rs41941615 | ns | I→V |
| C184 | 20 | 37238542 | TC | *NIPBL* | rs41942509 | ns | I→V |
| C185 | 22 | 5327792 | CT | *GADL1* | rs41992015 | ns | M→I |
| C186 | 22 | 5394424 | TC | *GADL1* | rs41991131 | ns | K→E |
| C187 | 26 | 39220494 | AT | *PRLHR* | rs109184180 | ns | F→L |
| C192 | 1 | 144601832 | GA | *PDE9A* |  | ns | V→M |
| C193 | 11 | 104011816 | AG | *NOTCH1* |  | ns | T→A |
| C194 | 20 | 31909476 | AT | *GHR* |  | ns | F→Y |
| C198 | 14 | 2708768 | GA | *CYP11B1* |  | ns | E→K |
| C199 | 14 | 1802265 | GA | *DGAT1* |  | ns | K→A |
| C202 | 5 | 60638414 | GA | *HAL* |  | ns | G→E |
| C204 | 11 | 104152101 | AG | *EGFL7* |  | ns | I→M |
| C206 | 14 | 2705205 | CT | *CYP11B1* |  | ns | A→V |
| C207 | 14 | 2465250 | AG | *RHPN1* | rs134458922 | ns | T→A |
| S1 | 5 | 60659116 | CT | *LTA4H* |  | ns | D→N |
| S8 | 14 | 3640627 | GC | *GPR20* | rs137068630 | ns | Q→H |
| P5 | 1 | 155284155 | TC | *RFTN1* |  | promoter |  |
| P9 | 3 | 80149065 | CA | *LEPR* |  | promoter |  |
| P10 | 5 | 75465669 | TC | *CACNG2* | rs110002002 | promoter |  |
| P11 | 11 | 103079381 | CA | *GTF3C5* | rs133114297 | promoter |  |
| P12 | 11 | 103325466 | CT | *GLT6D1* |  | promoter |  |
| P18 | 14 | 1508300 | CA | *RPL8* | rs110232042 | promoter |  |
| P23 | 14 | 1628421 | AG | *GPT* | rs133179781 | promoter |  |
| P24 | 14 | 1946673 | TA | *GPAA1* |  | promoter |  |
| P25 | 14 | 1957462 | GT | *OPLAH* | rs135089145 | promoter |  |
| P26 | 14 | 1969788 | CT | *SMPD5* |  | promoter |  |
| P29 | 14 | 2163044 | AG | *PUF60* | rs110924802 | promoter |  |
| P33 | 14 | 2221616 | TA | *LOC506831* |  | promoter |  |
| P34 | 14 | 2285869 | CT | *TSTA3* |  | promoter |  |
| P35 | 14 | 2311270 | TA | *EEF1D* | rs110716848 | promoter |  |
| P37 | 14 | 2327106 | CT | *NAPRT1* |  | promoter |  |
| P43 | 14 | 2832694 | AG | *SLURP1* | rs137564912 | promoter |  |
| P48 | 14 | 4129075 | TG | *EIF2C2* |  | promoter |  |
| P53 | 14 | 4187913 | CT | *CHRAC1* |  | promoter |  |
| P54 | 14 | 4188013 | AT | *CHRAC1* | rs110846620 | promoter |  |
| P58 | 14 | 4641787 | GA | *KCNK9* |  | promoter |  |
| P59 | 18 | 51527425 | TG | *GRIK5* |  | promoter |  |
| P62 | 18 | 51584422 | TC | *ATP1A3* | rs41886810 | promoter |  |
| P63 | 20 | 33027949 | CT | *PLCXD3* | rs42329885 | promoter |  |
| P68 | 20 | 35089819 | CT | *C9* | rs137340411 | promoter |  |
| P72 | 20 | 36634182 | AG | *GDNF* | rs110026134 | promoter |  |
| P75 | 26 | 42578252 | GA | *PLEKHA1* | rs136124582 | promoter |  |
| P78 | 11 | 110095540 | AC | *CACNA1B* |  | promoter |  |
| P86 | 5 | 80815103 | CG | *RABL4* |  | promoter |  |
| P89 | 14 | 2553525 | CA | *GPIHBP1* |  | promoter |  |
| P90 | 14 | 2314560 | CT | *EEF1D* |  | promoter |  |
| S13 | 8 | 30010071 | AC | *NFIB* |  | promoter |  |
| S14 | 8 | 30010037 | AT | *NFIB* |  | promoter |  |
| S15 | 6 | 30063427 | CT | *PDHA2* |  | promoter |  |
| S4 | 14 | 1677522 | GA | *CYHR1* |  | UTR |  |
| S11 | 14 | 4018378 | CA | *FAM135B* |  | UTR |  |
| S12 | 14 | 111990 | TC | *ZNF7* |  | UTR |  |
| U1 | 1 | 144604574 | GA | *PDE9A* |  | UTR |  |
| U2 | 1 | 144701204 | AG | *NDUFV3* | rs42137989 | UTR |  |
| U6 | 1 | 150153888 | TG | *CBR3* |  | UTR |  |
| U7 | 1 | 155383631 | GC | *DAZL* | rs42740179 | UTR |  |
| U14 | 3 | 42448014 | AG | *DPH5* |  | UTR |  |
| U18 | 3 | 58962715 | CT | *DDAH1* |  | UTR |  |
| U22 | 4 | 66346486 | TC | *MIR2419* | rs43400521 | UTR |  |
| U27 | 6 | 37679790 | CA | *PIGY* | rs110202238 | UTR |  |
| U28 | 6 | 38041449 | AG | *PKD2* |  | UTR |  |
| U31 | 6 | 38120968 | GT | *SPP1* | rs132812135 | UTR |  |
| U32 | 8 | 78871679 | TG | *SLC28A3* |  | UTR |  |
| U35 | 8 | 78872238 | CT | *SLC28A3* |  | UTR |  |
| U36 | 11 | 98393605 | TC | *STXBP1* | rs109831830 | UTR |  |
| U37 | 11 | 98406893 | CT | *PTRH1* |  | UTR |  |
| U38 | 11 | 103110243 | TC | *CEL* | rs134330946 | UTR |  |
| U39 | 11 | 105429329 | AG | *EHMT1* |  | UTR |  |
| U40 | 14 | 1118492 | AG | *H14C8ORF55* |  | UTR |  |
| U41 | 14 | 1155474 | CT | *LY6D* |  | UTR |  |
| U42 | 14 | 1449253 | CT | *LY6H* |  | UTR |  |
| U45 | 14 | 1489171 | GC | *C14H8orf33* | rs110097641 | UTR |  |
| U46 | 14 | 1694862 | CT | *VPS28* |  | UTR |  |
| U47 | 14 | 1806340 | CA | *HSF1* |  | UTR |  |
| U48 | 14 | 1907315 | TA | *LOC509113* | rs135705092 | UTR |  |
| U50 | 14 | 2019072 | CT | *GRINA* |  | UTR |  |
| U52 | 14 | 2462476 | TC | *RHPN1* | rs109416016 | UTR |  |
| U54 | 14 | 2565352 | GA | *LY6H* | rs110807190 | UTR |  |
| U57 | 16 | 44455788 | TG | *NMNAT1* | rs137536883 | UTR |  |
| U59 | 20 | 25247157 | TC | *ARL15* |  | UTR |  |
| U60 | 20 | 33229971 | CA | *PLCXD3* | rs137731223 | UTR |  |
| U63 | 20 | 35511396 | AG | *RICTOR* | rs41940571 | UTR |  |
| U64 | 23 | 34368798 | AG | *PRP4* | rs134097308 | UTR |  |
| U70 | 14 | 4743187 | CT | *KCNK9* | rs137422275 | UTR |  |
| U71 | 3 | 42465645 | TG | *SLC30A7* |  | UTR |  |

Additional file 1: Table S6. SNPs significantly associated with milk production traits in the Sequenom genotyped population

|  | SNP_ID | Traits | Maximum *P* value |
| --- | --- | --- | --- |
| 1 | U46 | All | 1.02E-04 |
| 2 | U48 | All | 3.34E-05 |
| 3 | S4 | MY,PY,FP,PP | 7.47E-06 |
| 4 | C113 | All | 1.18E-04 |
| 5 | C111 | All | 7.10E-05 |
| 6 | C199 | All | 2.06E-04 |
| 7 | P23 | MY,FY,FP,PP | 7.82E-05 |
| 8 | C110 | MY,PY,FP | 4.04E-05 |
| 9 | C109 | MY,FP | 3.06E-05 |
| 10 | C120 | MY,FP | 2.14E-04 |
| 11 | C133 | MY,FP,PP | 1.52E-04 |
| 12 | U41 | FP | 1.05E-08 |
| 13 | C194 | PP | 7.20E-08 |
| 14 | S12 | FP | 1.73E-07 |
| 15 | U42 | FP | 2.36E-07 |
| 16 | C128 | FP | 5.67E-07 |
| 17 | C140 | FP | 8.43E-07 |
| 18 | P18 | FP | 1.69E-06 |
| 19 | P89 | FP | 2.21E-06 |
| 20 | C138 | FP | 3.26E-06 |
| 21 | C112 | FP | 7.41E-06 |
| 22 | U47 | FP | 7.50E-06 |
| 23 | C127 | FP | 7.69E-06 |
| 24 | C132 | MY,FP | 7.08E-05 |
| 25 | C204 | PP | 2.08E-05 |
| 26 | C139 | FP | 2.29E-05 |
| 27 | P33 | FP | 2.36E-05 |
| 28 | P48 | FP | 2.55E-05 |
| 29 | C184 | PP | 3.01E-05 |
| 30 | P90 | FP | 4.25E-05 |
| 31 | C116 | FP | 6.99E-05 |
| 32 | N8 | FP | 7.60E-05 |
| 33 | C155 | PY | 8.26E-05 |
| 34 | S8 | FP | 9.16E-05 |
| 35 | P35 | FP | 1.05E-04 |
| 36 | C137 | FP | 1.15E-04 |
| 37 | C119 | FP | 1.31E-04 |
| 38 | C115 | FP | 1.45E-04 |
| 39 | C206 | FP | 1.63E-04 |
| 40 | C100 | PP | 2.05E-04 |

Additional file 1: Table S7 SNPs significantly associated with milk production traits in the combined population (Sequenom genotyped + imputed population)

|  | SNP_ID | MAF | Trait | *P* value |
| --- | --- | --- | --- | --- |
| 1 | C109 | 0.3352 | MY | 9.46E-13 |
|  |  |  | PY | 7.04E-10 |
|  |  |  | FP | 2.41E-25 |
|  |  |  | PP | 1.74E-04 |
| 2 | C110 | 0.2044 | MY | 7.00E-21 |
|  |  |  | FY | 2.39E-07 |
|  |  |  | PY | 5.65E-12 |
|  |  |  | FP | 1.16E-51 |
|  |  |  | PP | 3.94E-13 |
| 3 | C111 | 0.1733 | MY | 7.69E-22 |
|  |  |  | FY | 2.05E-12 |
|  |  |  | PY | 1.40E-10 |
|  |  |  | FP | 9.89E-67 |
|  |  |  | PP | 3.97E-18 |
| 4 | C112 | 0.4165 | FP | 2.30E-11 |
| 5 | C113 | 0.1644 | MY | 1.36E-22 |
|  |  |  | FY | 2.66E-10 |
|  |  |  | PY | 2.89E-11 |
|  |  |  | FP | 8.58E-63 |
|  |  |  | PP | 7.87E-18 |
| 6 | C114 | 0.2382 | MY | 1.27E-06 |
|  |  |  | FP | 4.19E-14 |
|  |  |  | PP | 1.94E-06 |
| 7 | C115 | 0.2538 | MY | 4.39E-07 |
|  |  |  | FP | 1.32E-16 |
|  |  |  | PP | 1.59E-08 |
| 8 | C116 | 0.2546 | MY | 3.13E-07 |
|  |  |  | FP | 3.63E-17 |
|  |  |  | PP | 9.61E-09 |
| 9 | C119 | 0.2432 | MY | 2.97E-06 |
|  |  |  | FP | 1.25E-14 |
|  |  |  | PP | 4.37E-07 |
| 10 | C120 | 0.22 | MY | 1.43E-15 |
|  |  |  | FY | 1.30E-08 |
|  |  |  | PY | 1.66E-07 |
|  |  |  | FP | 7.53E-46 |
|  |  |  | PP | 1.84E-13 |
| 11 | C125 | 0.0273 | PP | 9.31E-05 |
| 12 | C127 | 0.4698 | MY | 1.50E-04 |
|  |  |  | FY | 1.08E-04 |
|  |  |  | FP | 2.75E-15 |
|  |  |  | PP | 8.73E-09 |
| 13 | C128 | 0.4668 | MY | 2.12E-04 |
|  |  |  | FY | 9.03E-06 |
|  |  |  | FP | 3.45E-17 |
|  |  |  | PP | 1.35E-08 |
| 14 | C130 | 0.1399 | PY | 2.04E-06 |
| 15 | C132 | 0.3206 | MY | 7.49E-11 |
|  |  |  | PY | 6.77E-06 |
|  |  |  | FP | 4.77E-21 |
|  |  |  | PP | 1.51E-08 |
| 16 | C133 | 0.321 | MY | 1.01E-10 |
|  |  |  | PY | 8.05E-06 |
|  |  |  | FP | 1.65E-21 |
|  |  |  | PP | 1.77E-08 |
| 17 | C136 | 0.3791 | FP | 1.57E-09 |
| 18 | C137 | 0.3998 | MY | 7.22E-05 |
|  |  |  | FP | 7.57E-14 |
|  |  |  | PP | 5.48E-07 |
| 19 | C138 | 0.257 | MY | 4.00E-05 |
|  |  |  | FY | 3.16E-05 |
|  |  |  | FP | 2.34E-17 |
| 20 | C139 | 0.4212 | MY | 1.13E-09 |
|  |  |  | PY | 3.45E-07 |
|  |  |  | FP | 2.19E-21 |
| 21 | C140 | 0.3176 | MY | 1.29E-04 |
|  |  |  | FY | 4.87E-05 |
|  |  |  | FP | 8.93E-16 |
| 22 | C147 | 0.2358 | MY | 2.88E-06 |
|  |  |  | FP | 6.50E-11 |
|  |  |  | PP | 1.24E-04 |
| 23 | C163 | 0.1553 | MY | 4.24E-06 |
|  |  |  | FP | 3.34E-05 |
|  |  |  | PP | 2.24E-13 |
| 24 | C168 | 0.2388 | MY | 2.53E-06 |
|  |  |  | FP | 8.33E-05 |
|  |  |  | PP | 6.83E-17 |
| 25 | C169 | 0.347 | PP | 2.81E-09 |
| 26 | C171 | 0.2073 | FP | 1.88E-05 |
|  |  |  | PP | 1.31E-11 |
| 27 | C175 | 0.452 | PP | 1.56E-05 |
| 28 | C176 | 0.4784 | PP | 5.21E-05 |
| 29 | C184 | 0.2348 | FP | 1.97E-06 |
|  |  |  | PP | 5.79E-12 |
| 30 | C194 | 0.2329 | MY | 6.44E-09 |
|  |  |  | FP | 4.70E-14 |
|  |  |  | PP | 9.76E-38 |
| 31 | C198 | 0.041 | FP | 1.87E-05 |
| 32 | C199 | 0.1786 | MY | 4.05E-21 |
|  |  |  | FY | 3.76E-11 |
|  |  |  | PY | 2.96E-10 |
|  |  |  | FP | 3.04E-62 |
|  |  |  | PP | 1.43E-17 |
| 33 | C204 | 0.3274 | PP | 2.71E-07 |
| 34 | C206 | 0.4007 | FP | 1.57E-10 |
| 35 | C207 | 0.2754 | FP | 1.37E-12 |
|  |  |  | PP | 2.29E-04 |
| 36 | C30 | 0.07 | PP | 4.88E-06 |
| 37 | C8 | 0.4795 | PP | 1.01E-05 |
| 38 | N10 | 0.2386 | MY | 8.41E-07 |
|  |  |  | FP | 1.38E-13 |
|  |  |  | PP | 2.31E-06 |
| 39 | N7 | 0.2855 | PP | 4.68E-05 |
| 40 | N8 | 0.4814 | FY | 7.98E-05 |
|  |  |  | FP | 8.59E-11 |
| 41 | P18 | 0.4814 | MY | 2.62E-07 |
|  |  |  | PY | 6.63E-05 |
|  |  |  | FP | 2.26E-15 |
| 42 | P23 | 0.2037 | MY | 1.77E-20 |
|  |  |  | FY | 8.08E-08 |
|  |  |  | PY | 1.57E-11 |
|  |  |  | FP | 2.32E-52 |
|  |  |  | PP | 2.40E-13 |
| 43 | P24 | 0.239 | MY | 1.15E-06 |
|  |  |  | FP | 1.93E-13 |
|  |  |  | PP | 2.52E-06 |
| 44 | P25 | 0.2405 | MY | 1.48E-06 |
|  |  |  | FP | 1.34E-13 |
|  |  |  | PP | 7.93E-07 |
| 45 | P29 | 0.3032 | FP | 5.29E-06 |
| 46 | P33 | 0.4636 | FY | 2.29E-04 |
|  |  |  | FP | 7.91E-14 |
|  |  |  | PP | 6.93E-08 |
| 47 | P35 | 0.3819 | MY | 9.23E-06 |
|  |  |  | FP | 2.07E-15 |
|  |  |  | PP | 1.26E-07 |
| 48 | P48 | 0.2371 | MY | 1.62E-04 |
|  |  |  | FY | 7.24E-05 |
|  |  |  | FP | 1.54E-15 |
| 49 | P63 | 0.4374 | FP | 1.96E-04 |
|  |  |  | PP | 9.66E-11 |
| 50 | P72 | 0.1623 | FP | 4.81E-07 |
|  |  |  | PP | 2.48E-12 |
| 51 | P89 | 0.4905 | MY | 8.57E-11 |
|  |  |  | PY | 2.17E-08 |
|  |  |  | FP | 5.00E-18 |
| 52 | P90 | 0.2834 | MY | 1.21E-04 |
|  |  |  | FP | 2.88E-13 |
|  |  |  | PP | 6.81E-06 |
| 53 | S12 | 0.4816 | MY | 1.89E-07 |
|  |  |  | PY | 5.63E-05 |
|  |  |  | FP | 1.27E-15 |
| 54 | S4 | 0.1743 | MY | 1.17E-22 |
|  |  |  | FY | 3.14E-08 |
|  |  |  | PY | 1.16E-12 |
|  |  |  | FP | 3.30E-57 |
|  |  |  | PP | 7.71E-15 |
| 55 | S8 | 0.3014 | MY | 8.90E-05 |
|  |  |  | FP | 4.40E-13 |
|  |  |  | PP | 3.95E-06 |
| 56 | U36 | 0.1898 | PP | 1.43E-05 |
| 57 | U41 | 0.3815 | MY | 9.62E-05 |
|  |  |  | FY | 5.42E-09 |
|  |  |  | FP | 1.48E-23 |
|  |  |  | PP | 3.36E-11 |
| 58 | U42 | 0.4814 | MY | 2.02E-07 |
|  |  |  | PY | 5.84E-05 |
|  |  |  | FP | 1.67E-15 |
| 59 | U46 | 0.1674 | MY | 3.31E-22 |
|  |  |  | FY | 1.99E-09 |
|  |  |  | PY | 4.18E-11 |
|  |  |  | FP | 7.32E-60 |
|  |  |  | PP | 1.48E-17 |
| 60 | U47 | 0.4199 | FP | 2.95E-08 |
| 61 | U48 | 0.1661 | MY | 1.04E-22 |
|  |  |  | FY | 2.39E-10 |
|  |  |  | PY | 2.35E-11 |
|  |  |  | FP | 3.86E-63 |
|  |  |  | PP | 7.75E-18 |
| 62 | U50 | 0.4326 | FP | 6.36E-06 |
| 63 | U52 | 0.2204 | MY | 3.38E-06 |
|  |  |  | FP | 1.32E-13 |
|  |  |  | PP | 1.49E-05 |
| 64 | U60 | 0.3259 | PP | 7.47E-07 |
| 65 | U70 | 0.4413 | MY | 6.83E-05 |
|  |  |  | FP | 4.24E-09 |
| 66 | U71 | 0.0776 | PP | 1.13E-04 |

Additional file 1: Table S8 Relative expression of the 20 significant genes in mammary gland

| Gene | Expression level |
| --- | --- |
| *RPL8* | 16811.04±4545.76 |
| *EEF1D* | 6417.20±2742.50 |
| *VPS28* | 2660.75±4370.10 |
| *LOC786966* | 1046.39±175.14 |
| *HSF1* | 456.69±145.27 |
| *EIF2C2* | 450.16±212.10 |
| *NIPBL* | 446.66±274.36 |
| *PTK2* | 395.62±251.64 |
| *TRAPPC9* | 379.70±151.42 |
| *FAM83H* | 323.42±252.37 |
| *LOC509113* | 292.83±46.02 |
| *GHR* | 183.60±58.23 |
| *ZC3H3* | 147.66±185.98 |
| *HEATR7A* | 101.27±19.55 |
| *CPSF1* | 96.18±38.59 |
| *DGAT1* | 47.53±13.92 |
| *CYHR1* | 35.91±20.51 |
| *LOC506831* | 12.12±13.38 |
| *GPIHBP1* | 11.82±9.33 |
| *GPT* | 3.85±1.28 |

Additional file 1: Table S9 Primers designed for Sanger sequencing of *NOTCH1*, *PDE9A* and *GHR*

| Primer name | Forward | Reverse | Product Size |
| --- | --- | --- | --- |
| NOTCH1-1 | CGGAAGGAGAGGGCAGTAG | TTCAAAGCCGACTTCAAACC | 456 |
| NOTCH1-2 | GTCATCCCAGCGTTTCTTTC | CTCCACACGCAGCATAATTG | 571 |
| NOTCH1-3 | AATGACCCCCACTCTGGTC | GGAAACTGACACCTCCAAGG | 598 |
| NOTCH1-4 | GTGGTCACTTAGCCCCTGAG | TGTCACAGCAGACCTGTCCT | 725 |
| NOTCH1-5 | GCCCAGAGTAGGGTGTCCT | CACGTCTGTGCTGCTCCAT | 417 |
| NOTCH1-6 | GCTGGAGGAAGTCAGGAAGA | GCTATTCCAGGAGGCTCACA | 491 |
| NOTCH1-7 | TGTGAGCCTCCTGGAATAGC | TCGGACTACGTCCAACAGGT | 384 |
| NOTCH1-8 | CTGTTGGACGTAGTCCGATG | CCCCAGAGACCTAGCTCAAG | 451 |
| NOTCH1-9 | CGGTTATCTGGGGAGACTTG | GCCCAGCTTTGTCTGCTG | 391 |
| NOTCH1-10 | CAAGGATACGGGCGTTTG | CCTCCATACTGAGGGGTCAC | 369 |
| NOTCH1-11 | GTCTCTGACCCCAGGCAGT | GTCCTGAGACCCTGATCTGC | 660 |
| NOTCH1-12 | CTCTCCTGCTCCTGGTGTCT | GGTCATACTCGGAGCTGAGG | 412 |
| NOTCH1-13 | TGACCACTGCTCTGAACTGG | AGGCAGTTGCATTTGTACCC | 660 |
| NOTCH1-14 | CTCAGGGCAGGGCTTTTC | CTGCACCTCAGCCACCTG | 401 |
| NOTCH1-15 | ACGTGCTTGGACGGTAGAAC | CTCCGAGCCTCCCTTTGT | 343 |
| NOTCH1-16 | AGGGTTGTCCTGTGGCACT | CAGTCGTCGATGTCCGTCT | 240 |
| NOTCH1-17 | AGGAGCTTGCAGCTTTGGT | AGGGCTCCTGCTTCTAATCC | 460 |
| NOTCH1-18 | CGGCATGGATGTACCTGCT | GCTGGGGTGCTCAGTGTG | 307 |
| NOTCH1-19 | TGTCCATTTCTGTGGACTGG | CAGCACACCTCCTGACCTC | 361 |
| NOTCH1-20 | CTGAGGTCAGGAGGTGTGCT | GCCCTCCCGATGAGTCTAGT | 389 |
| NOTCH1-21 | CCACTTGGGTCCTTCTGGTA | GACAGGCACTCGTTGACATC | 589 |
| NOTCH1-22 | GAGGGGGATGTCAACGAGT | ACTGTCTTGAGGGGAACACG | 497 |
| NOTCH1-23 | CTCCTTCCCTCCCGATTC | AGTTCTGCCAAGGGTCGTC | 634 |
| NOTCH1-24 | CCACAGATCGAGGAGACCTG | AAGACCACATTGGTGTGCAG | 729 |
| NOTCH1-25 | GACCAGGGCTGCAACAGT | CACTGGGAGGATGACTGGAC | 698 |
| NOTCH1-26 | ATCCATCAGGGTGACTCCAC | AACTCTGGATGGCGTGACA | 436 |
| NOTCH1-27 | GCTGGTGCAGGAGAGAATTT | GAGGGGAGGTGCGACTAAG | 581 |
| NOTCH1-28 | CTGGAAGGGGAGGGAATCT | GGTCACCAGGTCAGGGAAG | 503 |
| NOTCH1-29 | GGAGCTTAGACGGATTGCAC | CAGACCAGGAAGGAGGGAAG | 511 |
| NOTCH1-30 | TGCGAACAATGACCATCCT | GGGGTCCACGTCTCTGTTT | 742 |
| NOTCH1-31 | GGGTCTCCATCTGTGTGGTT | TGCCATCTTGGGACTTCTTC | 686 |
| NOTCH1-32 | AAGGCTCGGAGGAAGAAGTC | GCGGCTGCATCTGTAAGTTC | 640 |
| NOTCH1-33 | AGCCGCAGCAGAACTTACAG | AAAAAGGCAGTGTTTCCATGT | 703 |
| PDE9A-1 | GAAGGAAAAGGAGGGGAGGT | AACGTCGGAACTCAGCCTAA | 599 |
| PDE9A-2 | TGCATGTAGCATTCCAGAGC | AGGTGCAAGGTGAGTCCATC | 365 |
| PDE9A-3 | GAGCCTCCCAACTCAACAAA | ACTTGCTCAAGGGGTCAATG | 706 |
| PDE9A-4 | CCAAGTCAAAGTGGGAGAGC | CAGCGTCTGCTAGGTTGTCA | 581 |
| PDE9A-5 | CATGAAAGCGTCCCGTATTT | AGAGCCCCTGAGGACATTCT | 603 |
| PDE9A-6 | GGTCTTGGACAATACACCTTCG | TTCGTTCTCAGCCTCACCTC | 400 |
| PDE9A-7 | TAACCCCGTGTTTCTCTTGG | CGCTTCCTCTCTTCTTCTGC | 356 |
| PDE9A-8 | CCATCAACTCTGGGGTTTGT | TGGCAGTAAGCTGTGGTCTG | 444 |
| PDE9A-9 | GGTCAGTCCTGAAGCACTCC | TCACATGCTCCTTGGTTCTG | 454 |
| PDE9A-10 | TGACTTCTAACCCCGACTGC | CGCAGGATTCTCACAGGAGT | 449 |
| PDE9A-11 | AGGAAGCAGGAATGCAGTTG | CCCCCAGATTCTCCCTAAAC | 364 |
| PDE9A-12 | AGGGTAGGTCTGGGGAACAT | GGAAGGCTGCATCCTACAGA | 474 |
| PDE9A-13 | TCATTGGTGGTGTCCACTGT | CCTTGTCTGTGTCAGCCTCA | 705 |
| PDE9A-14 | TTGGCATGAGGTCTGAATGA | CAGGAGAAAGGGTCCTACTGG | 417 |
| PDE9A-15 | GAGGGGTCACAGCAAAGTTC | CCTGCCTTGTGTGTCTCAAA | 416 |
| PDE9A-16 | CTCACGACCATCTGTGATGC | TGGGAGTGTGTGTGTGTGTG | 435 |
| PDE9A-17 | GAAACAGCGGGACTCAGAAC | GCTCCCTGAGGATAGAACACC | 451 |
| PDE9A-18 | GAAGGTAGACCGTGCAGAGC | AGGATGAGATGGTTGGATGG | 401 |
| GHR-1 | CTCAGCAATGGGAAGAGGAG | CCCAAGTCACCAGGACAAAA | 351 |
| GHR-2 | GGGAGGGAGGAAGAGAGAGA | CAAGGAGGGAGGGAGGAATA | 448 |
| GHR-3 | CATTCCTGCTTCAGCCGTAT | ATCCCCAGAGATGTCCTCCT | 348 |
| GHR-4 | GCATGAGACCAGAGGCAACT | ATTATCGCCAAAGGCAGAAA | 338 |
| GHR-5 | GGACCATCCATTACCCTCCT | TCACTGCCATATTTCCAGCA | 381 |
| GHR-6 | AGCTTGCTTTGAATGGCACT | TTGTTCGCCAGCTTATCTCA | 396 |
| GHR-7 | CTCTGCCATAAAGTGGAAGTGT | GAGGGGTTGTTGAACACAAAA | 390 |
| GHR-8 | AATGCAGAAGCACCTCATGG | TGAACTCCAGGTCCCTCATC | 381 |
| GHR-9 | CAAAAGGATCTGAAGAAGTGGA | ACGTTTCACTGGGTTGATGA | 415 |
| GHR-10 | TTTGCCTCATCATTCACTGC | GGAGGAGGTGAGCTTCATGT | 472 |
| GHR-11 | GAGGAGGGATGGTGTGATGT | AGGGGCAGCATCATTAGAAG | 647 |
| GHR-12 | GCAAAGGATGACGACTCTGG | GCTCACATAGCCACATGATGA | 753 |
| GHR-13 | CCACCACAGAAAGCCTTACC | AACTGCAAAAGCCACCTGAT | 591 |

Additional file 1: Table S10 Distribution of daughters in 30 sire families for association analysis

| Sire No. | No. of daughters | Sire No. | No. of daughters |
| --- | --- | --- | --- |
| 11101906 | 20 | 11199095 | 23 |
| 11103543 | 21 | 2302290 | 21 |
| 11101930 | 20 | 11101927 | 44 |
| 11102129 | 22 | 11196019 | 24 |
| 11199995 | 21 | 11196046 | 25 |
| 11101902 | 19 | 15198201 | 25 |
| 11101907 | 19 | 31101006 | 25 |
| 11102912 | 22 | 31103406 | 23 |
| 11102910 | 22 | 31103409 | 25 |
| 11102909 | 20 | 31195510 | 26 |
| 11102919 | 23 | 31195513 | 25 |
| 97314 | 20 | 31196415 | 26 |
| 11100113 | 45 | 31197201 | 29 |
| 11101916 | 19 | 31198202 | 27 |
| 11101929 | 28 | 31199003 | 25 |

Additional file 1: Table S11 Primers for real time RT-PCR of 20 significant genes

| Gene | Forward | Reverse | Product size |
| --- | --- | --- | --- |
| *EEF1D* | GTGGTGGAGGATGACAAGGT | TGTTGAAAGCAGCAATGTCG | 97 |
| *RPL8* | TCGTGTGTTGTCTGGAGGAG | TTGGCAGAGGAGATGACCTT | 151 |
| *VPS28* | TCCTCTCTTTCCTGGTCACCT | GTCGTACTTCTCCCGCTCAC | 149 |
| *GPIHBP1* | AGGACGAGGAGGAAGAGGAG | AGTGTTCCAGGAGGAGACGA | 161 |
| *ZC3H3* | TCAGTGAGACTCCGCTCTCC | CGCAGGCTGAACTGACTCTT | 139 |
| *CPSF1* | CAGGATGAGGTGGACGAGAT | CAGGGTCCAATGTTGAGGAT | 110 |
| *CYHR1* | GCCGTTTTCACAACCAACTC | CAGCGACAGATGTCCTCCTT | 125 |
| *DGAT1* | GACACAGACAAGGACGGAGAC | AATTCAGGATGCCACGGTAG | 124 |
| *EIF2C2* | TCTCGGCAACAGCGTTCTAT | GCAGTGCGTTATCTCCACCT | 161 |
| *FAM83H* | GACAAGTGCCGAGTCAACCT | CACAATCCACCAGGAGGAAC | 133 |
| *GHR* | GCTCGCAGGTCCTACAGGT | TGACAAGGAAAGCTGGTGTG | 110 |
| *GPT* | AATGGGCTGAAGGAGAAGGT | CTCGGTGAAGGGCTTCTTTA | 147 |
| *HEATR7A* | TCTGACAACACCTGGGTCTG | GCTCCCTCTCCTCCTGGTAT | 192 |
| *HSF1* | AGAGAGGGACGACACCGAGT | GAGTCCATGCTCTCCTGCTT | 192 |
| *LOC506831* | ACGACGTGCTGATGAAGGAC | GTCAAACACCAGGGGCTTC | 209 |
| *LOC509113* | TGAGAGTTTCACCAGCCACA | AAGGTGGGAGCATCAGGTC | 193 |
| *LOC786996* | CTGGTGAACATCAGGAACGAT | CTCCTTGGCTGTCATGTCCT | 138 |
| *NIPBL* | TGCCAGAAAATTCAGCTCCT | ACTTTTGCAGATTCGGATGG | 148 |
| *PTK2* | AAAGCAACAGTGAGCCAACC | ATCCAAATGGAGCCAGTGAA | 173 |
| *TRAPPC9* | TGTCAGGCACCTGTCCTTC | GTCTGGGAGGGTGAGGAGTT | 136 |

Additional file 1: Table S12 LD levels (*r*^2^) between SNPs on BTA14 and the DGAT1 SNP (C199) and *P* values of association analysis for fat percentage using models without and with C199 fixed

| SNP | *r*^2^ | *P* value  without C199 | *P* value  C199 fixed |
| --- | --- | --- | --- |
| C111 | 0.986 | 9.885E-67 | 5.244E-02 |
| U46 | 0.923 | 7.323E-60 | 2.889E-01 |
| C113 | 0.917 | 8.579E-63 | 7.052E-02 |
| U48 | 0.902 | 3.861E-63 | 5.951E-02 |
| S4 | 0.884 | 3.304E-57 | 2.783E-01 |
| P23 | 0.699 | 2.320E-52 | 1.812E-02 |
| C110 | 0.698 | 1.159E-51 | 2.662E-02 |
| C120 | 0.625 | 7.530E-46 | 8.078E-02 |
| C112 | 0.345 | 2.300E-11 | 2.094E-02 |
| C109 | 0.341 | 2.410E-25 | 4.571E-01 |
| U47 | 0.244 | 2.948E-08 | 9.149E-03 |
| C132 | 0.24 | 4.769E-21 | 6.166E-02 |
| C133 | 0.237 | 1.647E-21 | 4.243E-02 |
| C139 | 0.199 | 2.194E-21 | 1.500E-03 |
| U41 | 0.19 | 1.479E-23 | 7.155E-05 |
| P48 | 0.18 | 1.539E-15 | 3.747E-02 |
| C138 | 0.153 | 2.337E-17 | 1.273E-02 |
| P89 | 0.148 | 5.003E-18 | 5.665E-02 |
| P18 | 0.147 | 2.258E-15 | 7.627E-02 |
| S12 | 0.146 | 1.271E-15 | 6.440E-02 |
| U42 | 0.146 | 1.674E-15 | 7.054E-02 |
| C128 | 0.138 | 3.451E-17 | 3.265E-03 |
| U50 | 0.121 | 6.361E-06 | 7.642E-01 |
| P35 | 0.121 | 2.074E-15 | 1.164E-02 |
| C140 | 0.113 | 8.929E-16 | 1.378E-03 |
| C127 | 0.096 | 2.750E-15 | 1.230E-03 |
| P33 | 0.093 | 7.911E-14 | 5.613E-03 |
| N8 | 0.089 | 8.587E-11 | 1.099E-02 |
| P90 | 0.078 | 2.878E-13 | 3.698E-03 |
| S8 | 0.076 | 4.400E-13 | 8.109E-04 |
| C207 | 0.074 | 1.370E-12 | 1.136E-02 |
| C115 | 0.069 | 1.324E-16 | 1.891E-05 |
| C116 | 0.069 | 3.630E-17 | 8.241E-06 |
| C137 | 0.066 | 7.567E-14 | 5.601E-04 |
| P25 | 0.065 | 1.337E-13 | 4.976E-04 |
| C206 | 0.065 | 1.574E-10 | 1.202E-02 |
| P24 | 0.064 | 1.925E-13 | 7.891E-04 |
| C119 | 0.064 | 1.246E-14 | 1.400E-04 |
| N10 | 0.063 | 1.377E-13 | 6.176E-04 |
| U52 | 0.056 | 1.316E-13 | 3.203E-04 |
| C136 | 0.055 | 1.571E-09 | 4.005E-02 |
| C114 | 0.054 | 4.194E-14 | 2.343E-04 |
| U70 | 0.043 | 4.244E-09 | 8.000E-04 |
| C147 | 0.038 | 6.500E-11 | 8.279E-04 |
| C130 | 0.034 | 5.843E-02 | 1.222E-01 |
| C198 | 0.025 | 1.868E-05 | 9.498E-03 |
| C125 | 0.006 | 1.299E-02 | 4.226E-02 |
| P29 | 0.002 | 5.292E-06 | 8.715E-02 |
