# Supplementary material for: Targeted resequencing of GWAS loci reveals novel genetic variants for milk production traits
Source: BMC Genomics. 2014 Dec 15;15(1):1105. doi: 10.1186/1471-2164-15-1105 (PMC4377845; doi:10.1186/1471-2164-15-1105)
Supplement: Supplementary file 2 — Additional file 2: Figure S1: DNA sequencing chromatogram of SNPs detected in NOTCH1, PDE9A and GHR for validation of NGS results. Figure S2. Relative mRNA Expression of 20 significant genes in eight tissues of four cows by real time RT-PCR. Figure S3. Linkage disequilibrium levels (r 2) between the significant SNPs on BTA14 obtained by using Haploview. (DOC 10 MB) [file 12864_2014_6834_MOESM2_ESM.doc]

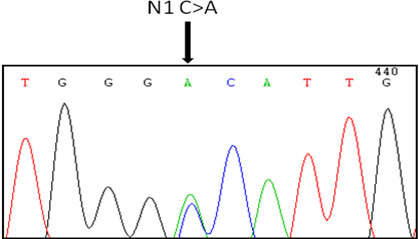

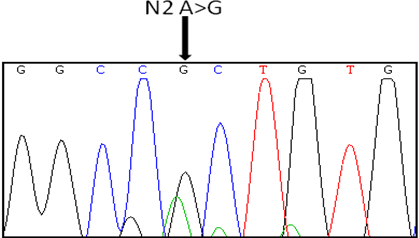


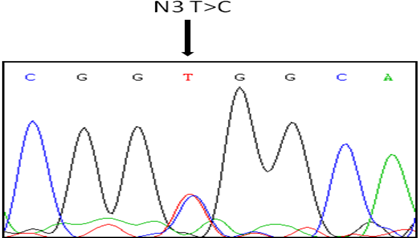

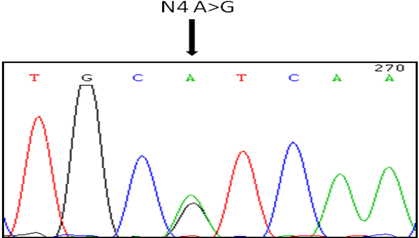


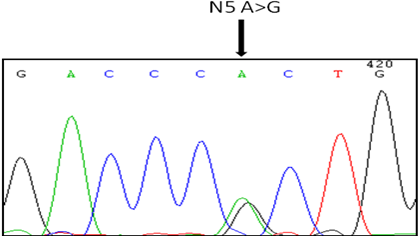

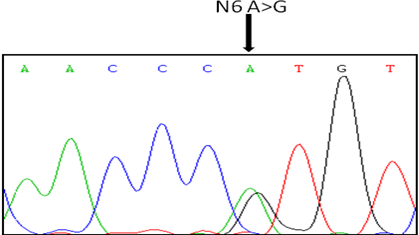


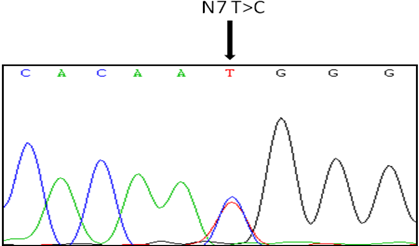

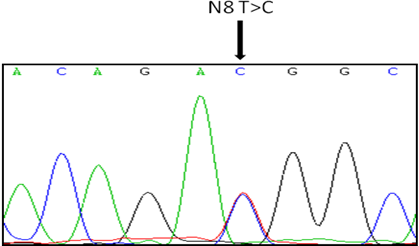


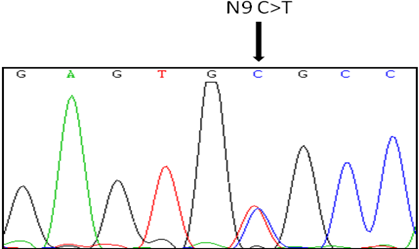

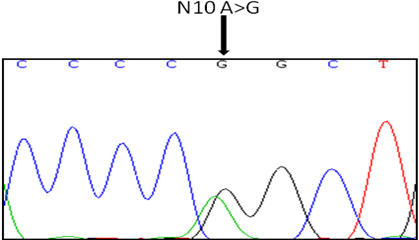


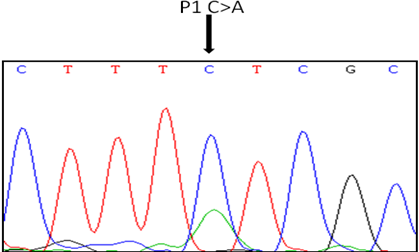

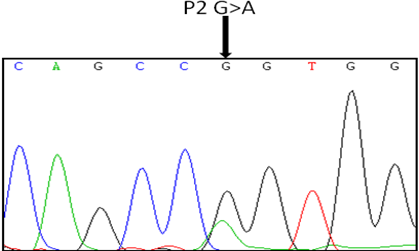


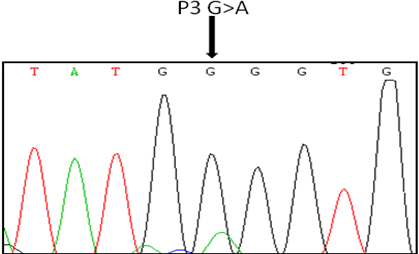

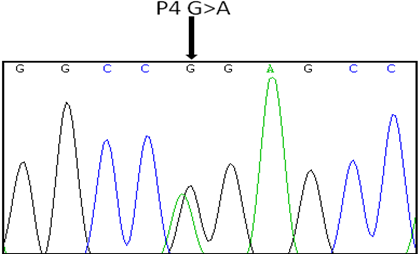


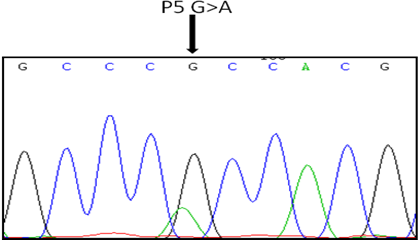

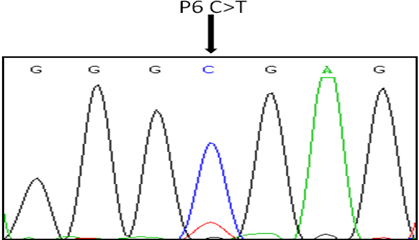


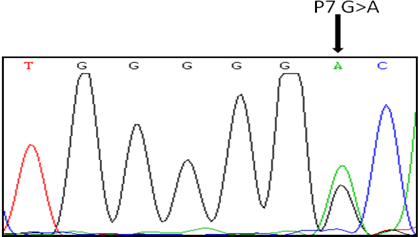

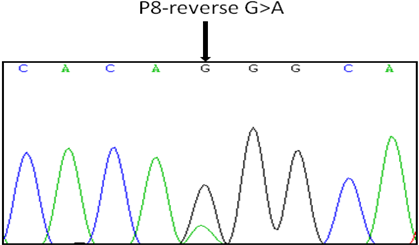


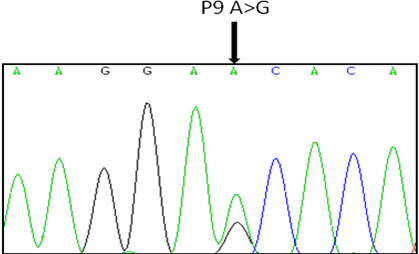

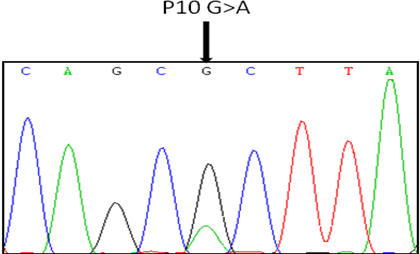


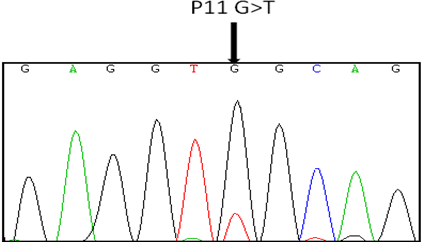

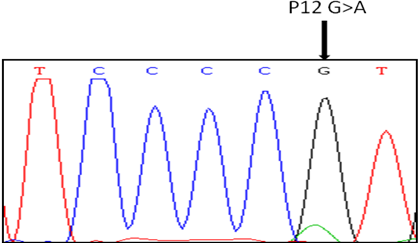


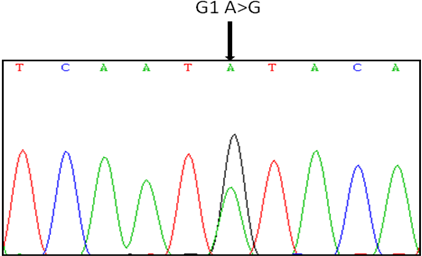

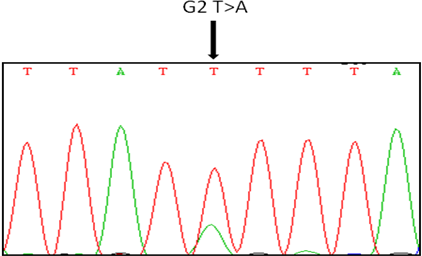


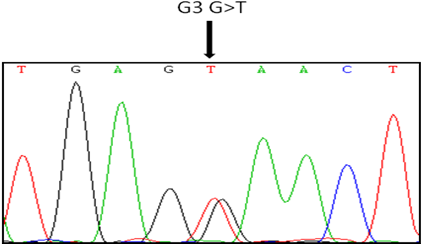

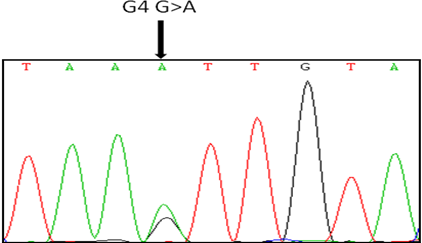


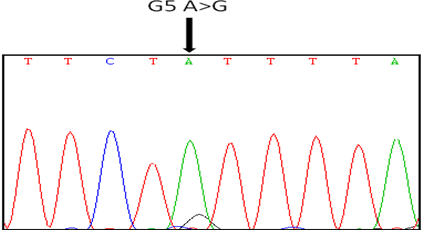

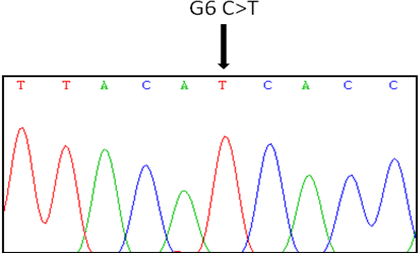


Additional file 2: Figure S1. DNA sequencing chromatogram of SNPs detected in *NOTCH1*, *PDE9A* and *GHR* for validation of NGS results


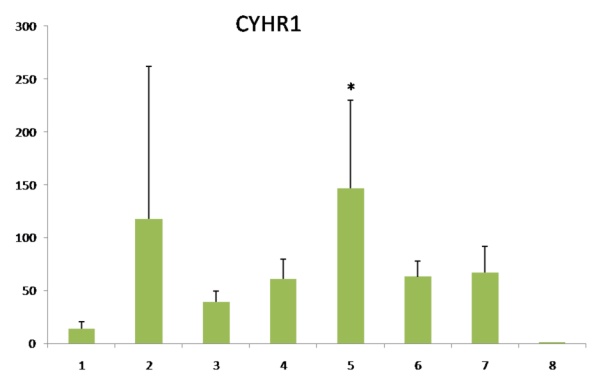

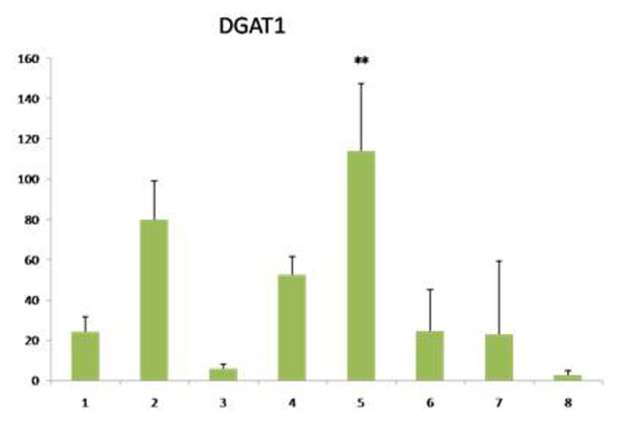


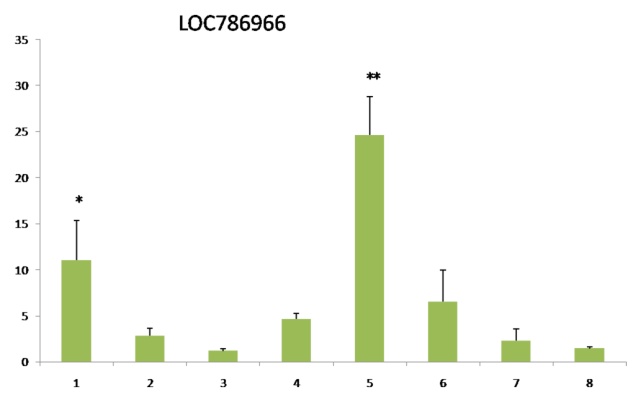

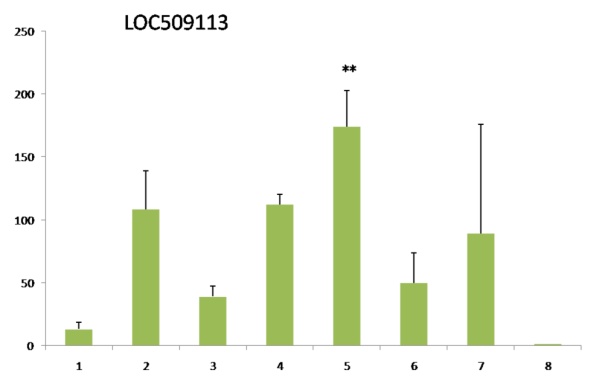


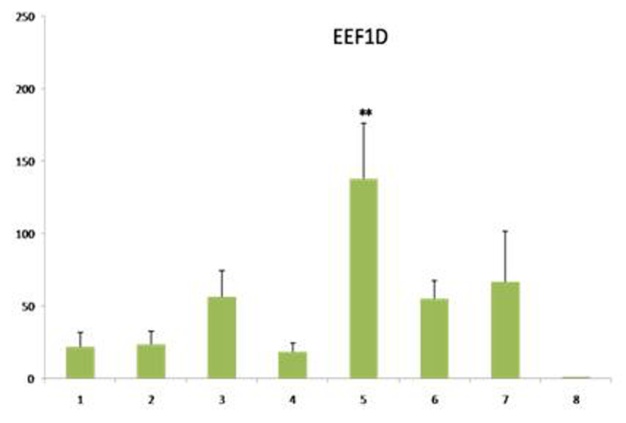

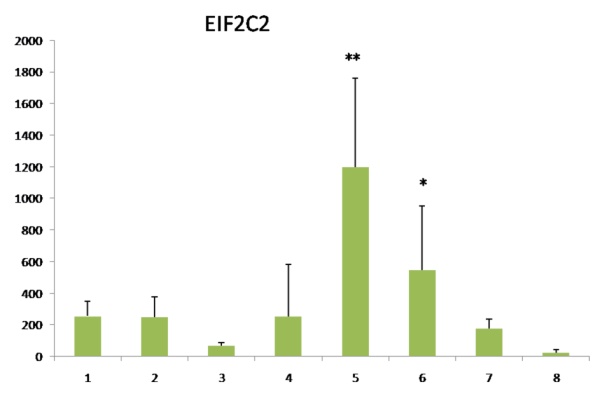


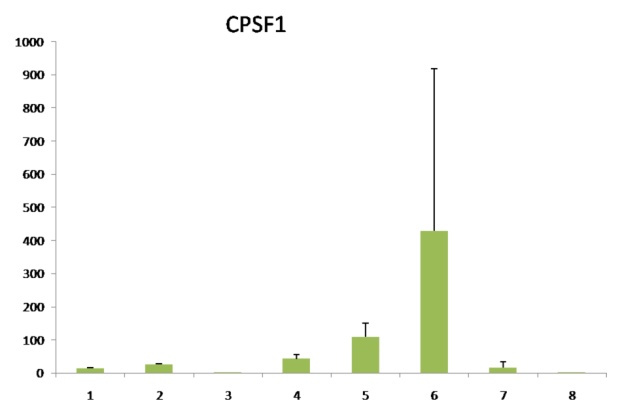

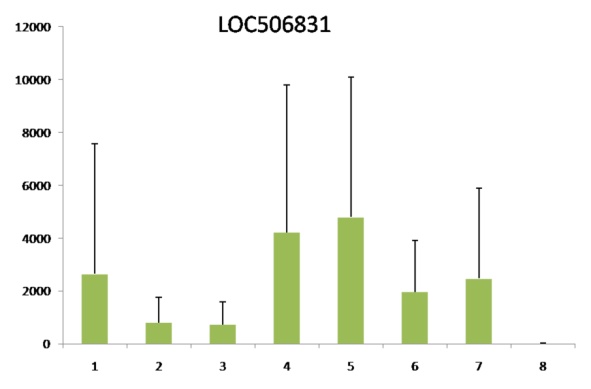


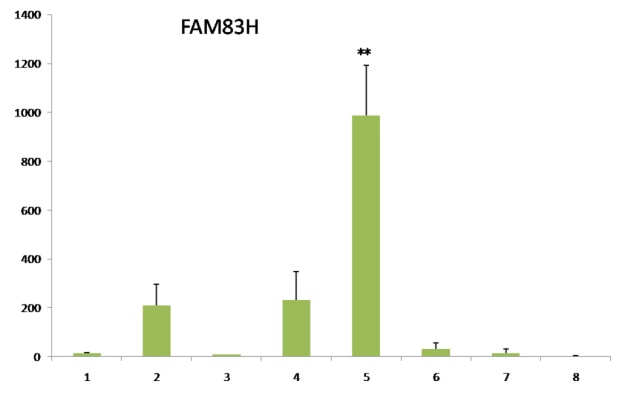

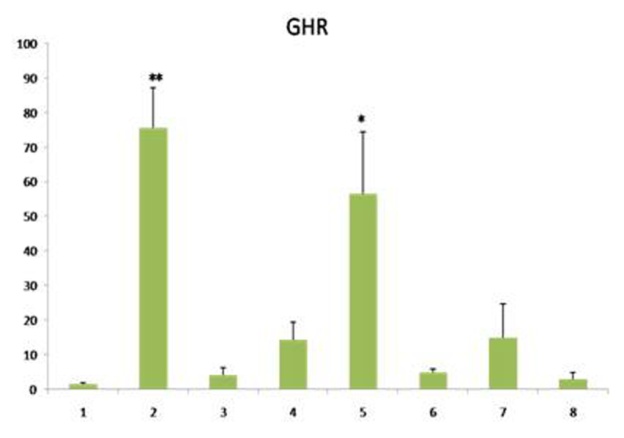


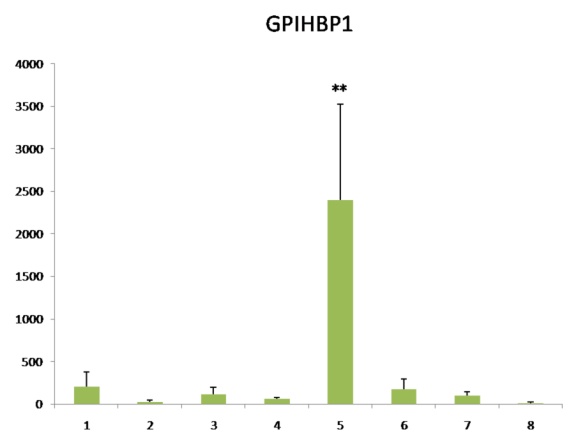

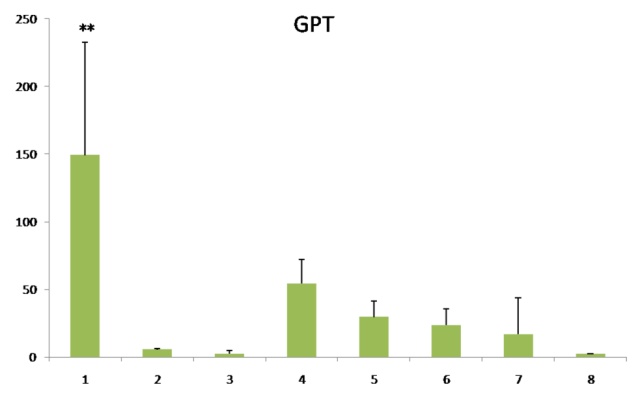


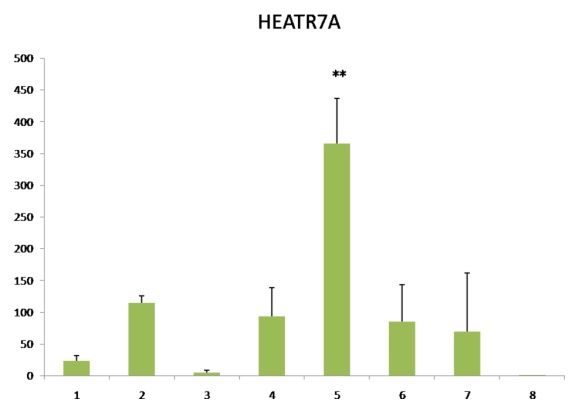

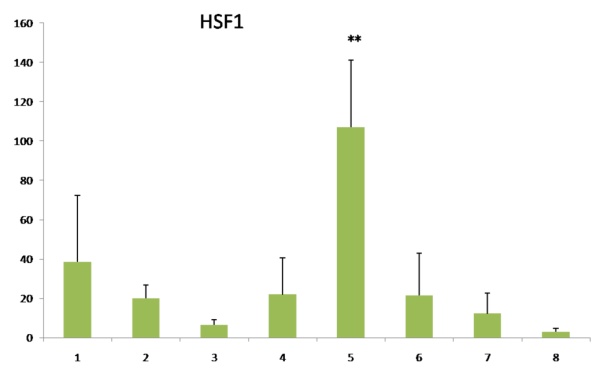


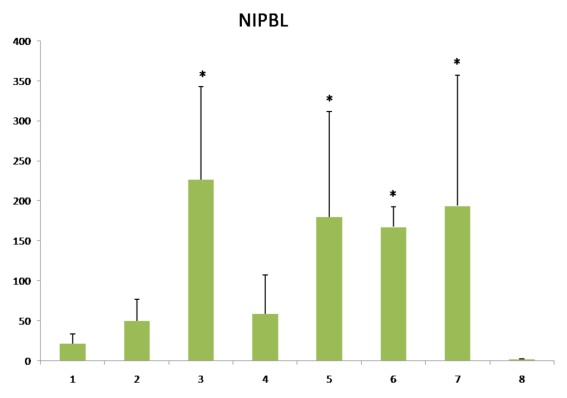

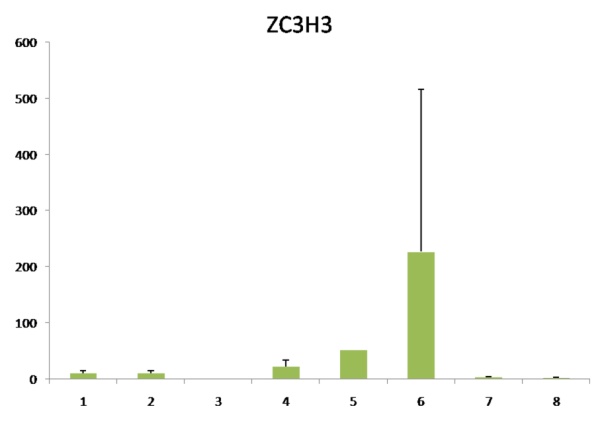


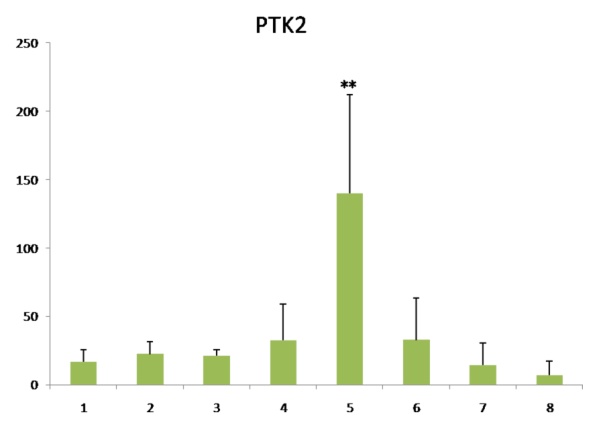

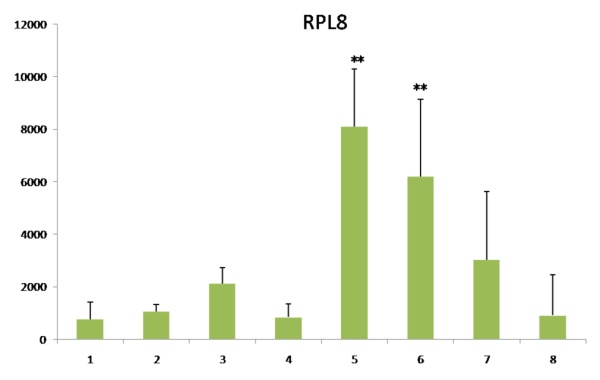


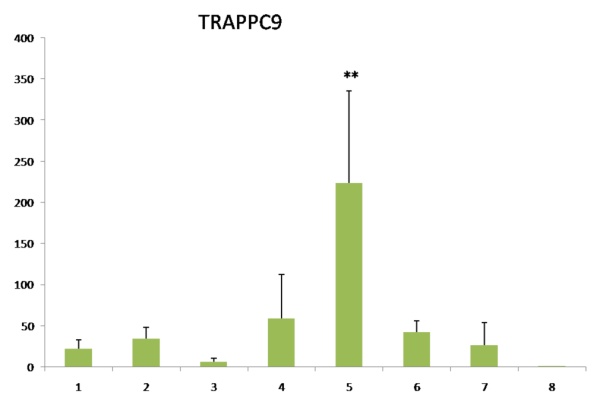

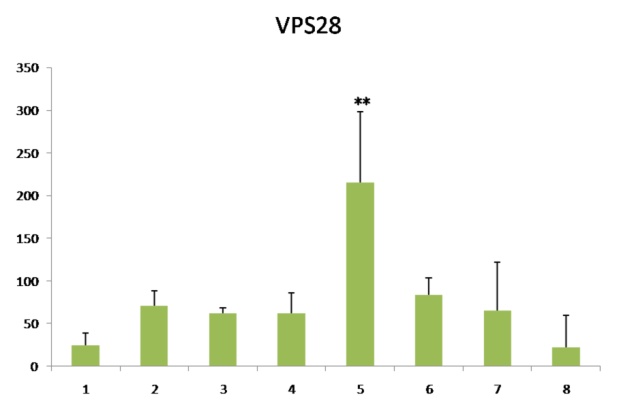


Additional file 2: Figure S2. Relative mRNA Expression of 20 significant genes in eight tissues of four cows by real time RT-PCR. Numbers (1~8) under the lateral axis represent heart, liver, lung, kidney, mammary, ovary, uterus and muscle, respectively. Three replicates for each reaction were performed.


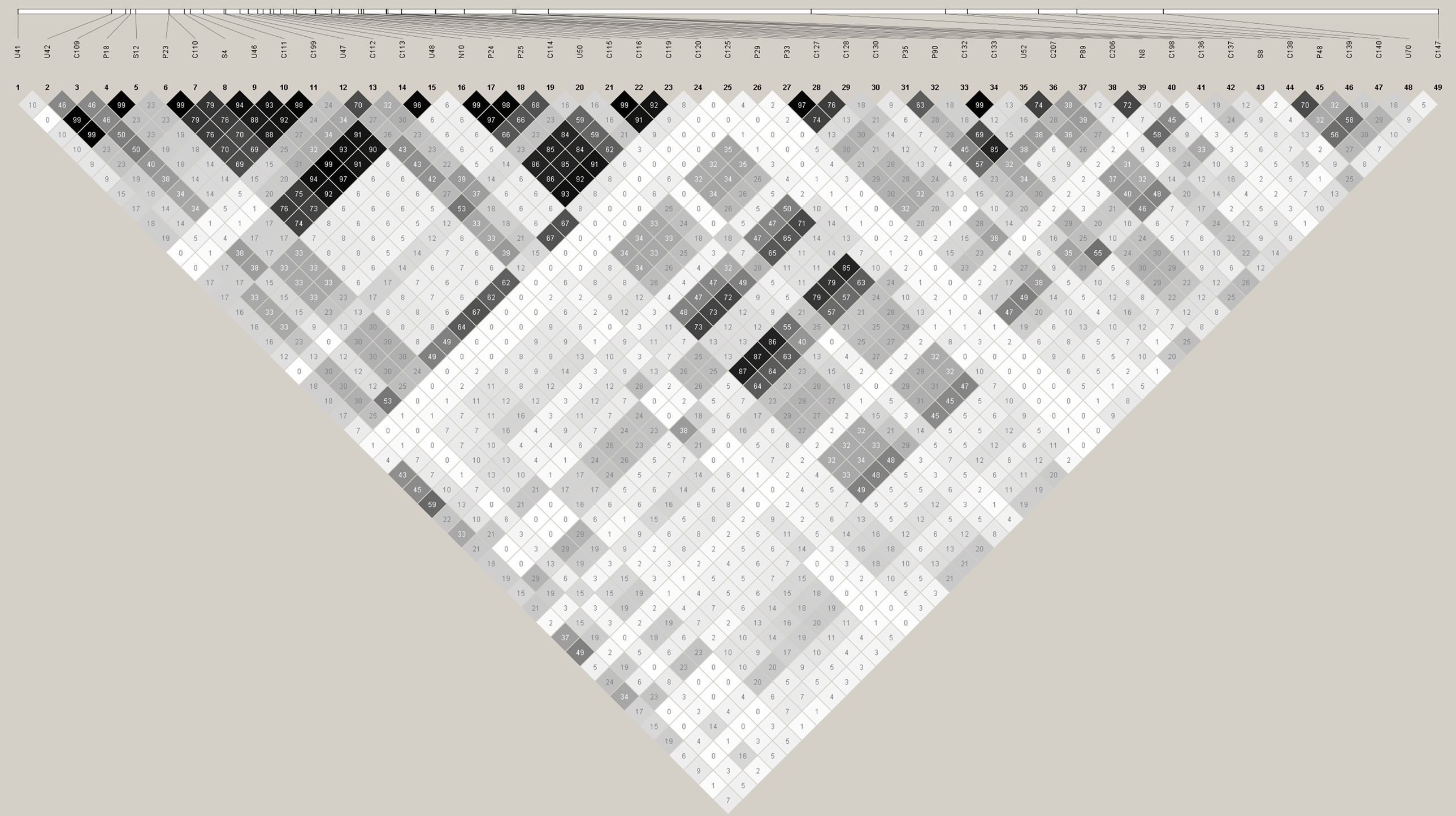


Additional file 2: Figure S3. Linkage disequilibrium levels (*r*^2^) between the significant SNPs on BTA14 obtained by using Haploview.
